# Contrasting patterns in diversity and community assembly of bacterioplankton and three size fractions of protists in the South China Sea

**DOI:** 10.1128/aem.00436-25

**Published:** 2025-06-26

**Authors:** Xinyi Zheng, Xin Guo, Xiaoqing Lin, Lingfeng Huang

**Affiliations:** 1 Key Laboratory of the Ministry of Education for Coastal and Wetland Ecosystems, College of the Environment and Ecology, Xiamen University 598522, Xiamen, China; 2 State Key Laboratory of Marine Environmental Science, College of Ocean and Earth Sciences, Fujian Key Laboratory of Marine Carbon Sequestration, Xiamen University 534813, Xiamen, China; 3 Carbon Neutral Innovation Research Center, Xiamen University 12466, Xiamen, China; University of Delaware, Lewes, Delaware, USA

**Keywords:** community assembly, marine microbes, diversity, South China Sea

## Abstract

The microbial food web plays a critical role in marine ecosystems, composed of various cell sizes of microbial organisms. Here, high-throughput sequencing of the 16S and 18S rRNA genes was conducted to detect the community structure and distribution patterns of bacterioplankton (0.2 µm–2 µm) and three size fractions of protist communities, i.e., pico-protist (0.2 µm–2 µm), nano-protist (2 µm–20 µm), and micro-protist (20 µm–200 µm), in the euphotic zone of the South China Sea. The trophic mode compositions of protist communities varied significantly across three size fractions, characterized by a substantial prevalence of parasitic pico-protists (40% amplicon sequence variants) and a greater predominance of mixotrophic taxa within nano- and micro-protist communities. Furthermore, we detected stronger vertical stratification of bacterial and pico-protist communities, corresponding to the wider niche breadth of smaller cells and reliance on passive dispersal. Additionally, both bacterial and protist community assemblies were dominated by stochastic processes. The relative contribution of homogeneous selection in nano-protist community assembly was greater compared to other size fractions, probably related to high relative abundance of mixotrophs. In summary, our results suggest that both cell size and trophic mode affect marine microbial community assembly, and that neither the “size-plasticity” hypothesis nor the “size-dispersal” hypothesis fully matched microbial communities. Our analyses are important for a better understanding of the assemblage processes of marine epipelagic microbial communities and how they will respond to global change.

**IMPORTANCE:**

Cell size is a key feature that influences microbial biology at both the cellular and community levels. Poorly understood is the extent to which diverse ecological factors influence the assembly of microbial communities of various sizes. Two important hypotheses addressing the mechanisms of biome assembly are “size-plasticity” and “size-dispersal.” Here, we investigated epipelagic microbial communities to reveal differences in the ecological functions of various microbial sizes, to explore the association of ecological processes with niche and cell size, and to expand the current understanding of marine microbial community assemblages and their possible responses to future global change.

## INTRODUCTION

The microbial food web plays a critical role in marine ecosystems as it dominates primary production and supports higher trophic levels, of which the structure is usually separated into several size classes, i.e., pico-, nano-, and micro-plankton (cell size of 0.2 µm–2 µm, 2 µm–20 µm, and 20 µm–200 µm, respectively) (1, 2). In recent years, the development of high-throughput sequencing technology and increasing global oceanic surveys have facilitated our knowledge of microbial diversity and biogeographic patterns (3, 4), discovering complex trophic modes, and further recognizing their important ecological functions under climate change (5, 6). Nevertheless, most studies have treated unicellular protists smaller than 200 µm as a single group without considering size-based distinctions (7, 8). As a result, differences in trophic pattern composition and environmental drivers among microorganisms of different sizes remain underexplored.

Cell size is a key characteristic that affects microbial biology at both cellular and community levels (9). It is proven that metabolic rate and population growth rate scale differently with body mass across different sizes of organisms from prokaryotes to unicellular protists to metazoans (10, 11). Cell size is also related to the environmental adaptability of plankton, as the sinking rate increases with the cell size of microorganisms, which affects their vertical distribution and further regulates aquatic food web organization (9, 12). In the euphotic zone, small cells (<2 µm), common in oligotrophic waters, tend to remain suspended due to slow sinking, promoting tight trophic coupling among autotrophs, bacteria, and microphages, and enhancing local recycling (13, 14). In contrast, large cells (>2 µm), typical in eutrophic conditions, contribute more to carbon export through faster sedimentation or incorporation into zooplankton fecal pellets (13, 14). Thus, nutrient supply has a critical role in determining a community’s size structure and element transfer and cycling in the pelagic ecosystem (12). Previous studies have suggested that environmental factors, spatial factors, and biotic interactions are strongly correlated with bacterial and protist distribution patterns in marine ecosystems (7, 15–17). However, the effect degree of different ecological factors on community assembly of microbes across different size fractions is poorly understood.

As a key topic in microbial ecology, increasing studies are devoted to unraveling the mechanisms that shape microbial biogeographic patterns (18–20). While dispersal limitations have been documented for a wide range of marine microorganisms at the regional scale (21), the relative contributions of stochastic processes (including dispersal, drift, and other unpredictable events) and deterministic processes (including environmental filtering and biotic interactions) in shaping patterns across different sizes and types of microorganisms remain underexplored (8). Two key hypotheses have been proposed for the community assembly mechanisms of multicellular organisms, the “size-plasticity” hypothesis and the “size-dispersal” hypothesis (22). These two hypotheses have also been applied to study community assembly mechanisms of unicellular microorganisms (22, 23). The “size-plasticity” hypothesis considers that larger organisms are less plastic in their fundamental niches and, thus, more probable to be selected by the environment during dispersal (23). The “size-dispersal” hypothesis suggests that larger organisms are more dispersal-limited due to lower passive transport capacity compared to smaller organisms (22, 24). In one study, Farjalla et al. (22) found environmental determinism increased with the body size of three groups of organisms (bacteria, zooplankton, and macroinvertebrates), consistent with the “size-plasticity” hypothesis. Another study of the spatial patterns of 12 aquatic organismal groups (ranging from bacteria to fish) in 99 farmland ponds revealed strengthened dispersal limitation with body size, which supported the “size-dispersal” hypothesis (25). Additionally, bacteria (0.2 µm–1.2 µm) were found to have less environmental filtering than protists (>1.2 µm) in the basin of the East China Sea (8). A study on a large scale of subtropic-tropic marginal seas revealed stronger dispersal limitation in nano-sized microbial flagellate communities compared to their pico-sized counterparts (26). There are also studies showing that the ecological processes controlling community structure depend not only on the size of organisms, but also on environmental conditions and spatial scales (27–29).

**Fig 1:**
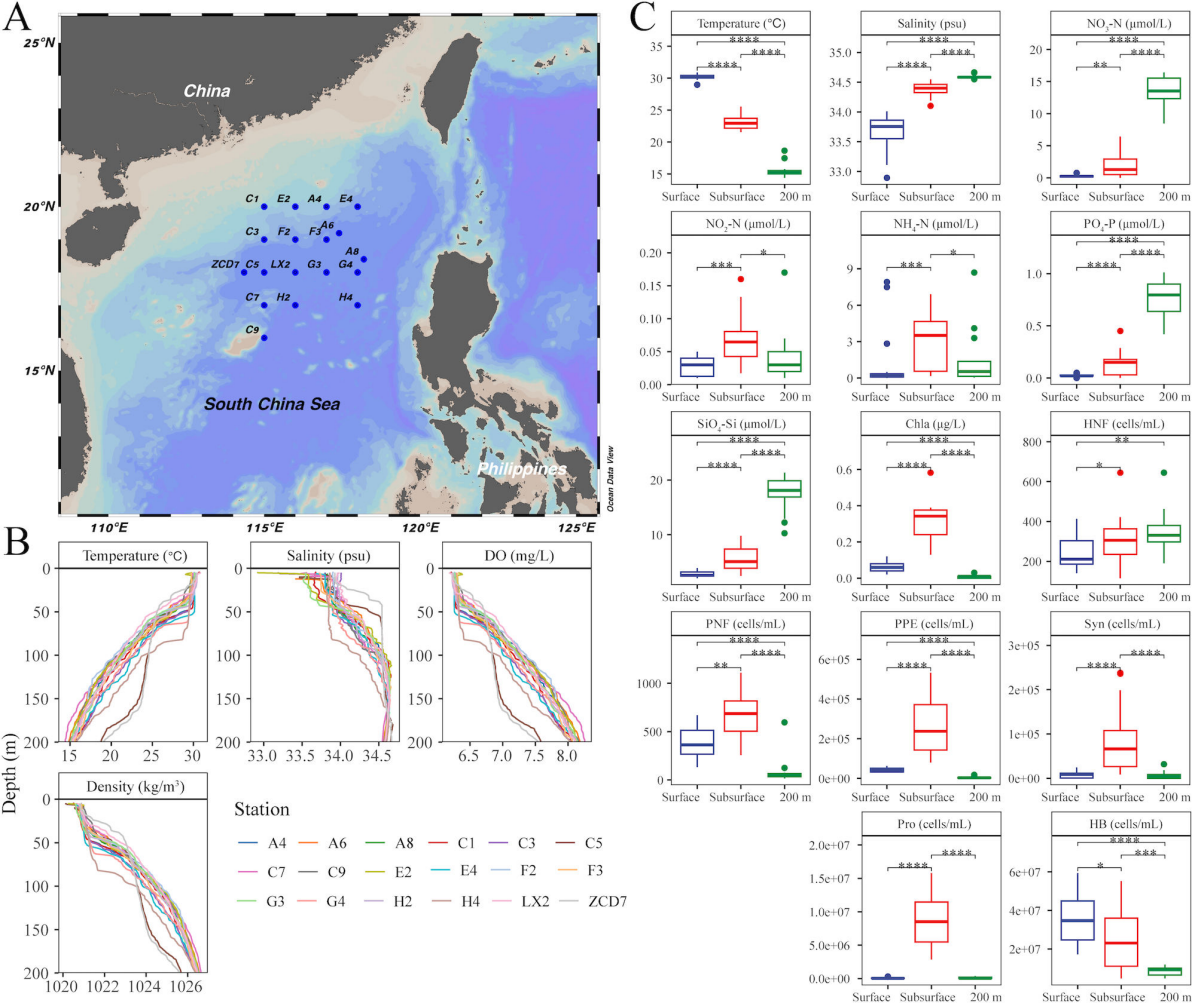
Overview of sampling area and environmental factors. (**A**) Location of 18 sampling stations (blue circles) in the South China Sea. Map is from Ocean Data View (Schlitzer, Reiner, Ocean Data View, https://odv.awi.de, 2025). (**B**) Water column profiles. (**C**) Environmental factors at different water layers. Asterisk indicates significant differences at the level of *P* < 0.05 (*, *P* < 0.05; **, *P* < 0.01; ***, *P* < 0.001; ****, *P* < 0.0001). The abbreviations of environmental variables are NO_3_-N, nitrate; NO_2_-N, nitrite; NH_4_-N, ammonium; PO_4_-P, phosphate; SiO_4_-Si, silicate; HNF, heterotrophic nano-sized flagellates; PNF, pigmented nano-sized flagellates; PPE, photosynthetic picoeukaryotes; Syn*, Synechococcus*; Pro, *Prochlorococcus*; HB, heterotrophic bacteria.

In this study, we aimed to better understand the biogeographic patterns of the major components of the microbial food web and to disentangle the relative importance of environmental and spatial factors in shaping the microbial communities with cell sizes ranging from 0.2 μm to 200 µm. To achieve this, we investigated bacterioplankton and pico-, nano-, and micro-protist communities from the euphotic zone of the South China Sea (SCS) (Fig. 1A) using 16S and 18S rRNA gene amplicon sequencing. We hypothesized that (i) the bacterial and three size fractions of protist communities would show different spatial distribution patterns across three water layer depths in the epipelagic SCS, and (ii) the driving mechanisms of the bacterial and three size fractions of protist communities would be distinct. If the “size-plasticity” hypothesis is true, it is expected that the relative importance of the deterministic process in community assembly increases with cell size. If the “size-dispersal” hypothesis is true, stochastic processes play a more important role in shaping communities of larger organisms.

## RESULTS

### Environmental parameters

The study area was located in the South China Sea basin area (Fig. 1A), where the mixed layer was at a depth of about 50 m, except that the depth at station H4 was up to 80 m (Fig. 1B). Sampling covered the surface layer (5 m), the subsurface layer under the mixed layer (75 m–90 m), and the bottom of the euphotic zone (200 m). Abiotic and biotic parameters showed significant differences among three water layers (Fig. S1), representing distinct environmental conditions. As shown in Fig. 1 and Table S1, the surface layer was warmer and nutrient-poor, the subsurface layer had higher concentrations of nitrite (NO_2_-N) and ammonium (NH_4_-N), and the bottom layer of the euphotic zone had higher concentrations of nitrate (NO_3_-N), phosphate (PO_4_-P), and silicate (SiO_4_-Si). The subsurface layer showed the highest abundance of pigmented nano-sized flagellates (PNFs), photosynthetic picoeukaryotes (PPE), *Prochlorococcus* (Pro), and *Synechococcus* (Syn). The average abundance of heterotrophic nano-sized flagellates (HNFs) increased with depth, while heterotrophic bacteria (HB) showed the opposite trend.

### Taxonomic and trophic mode compositions of microbial communities

**Fig 2:**
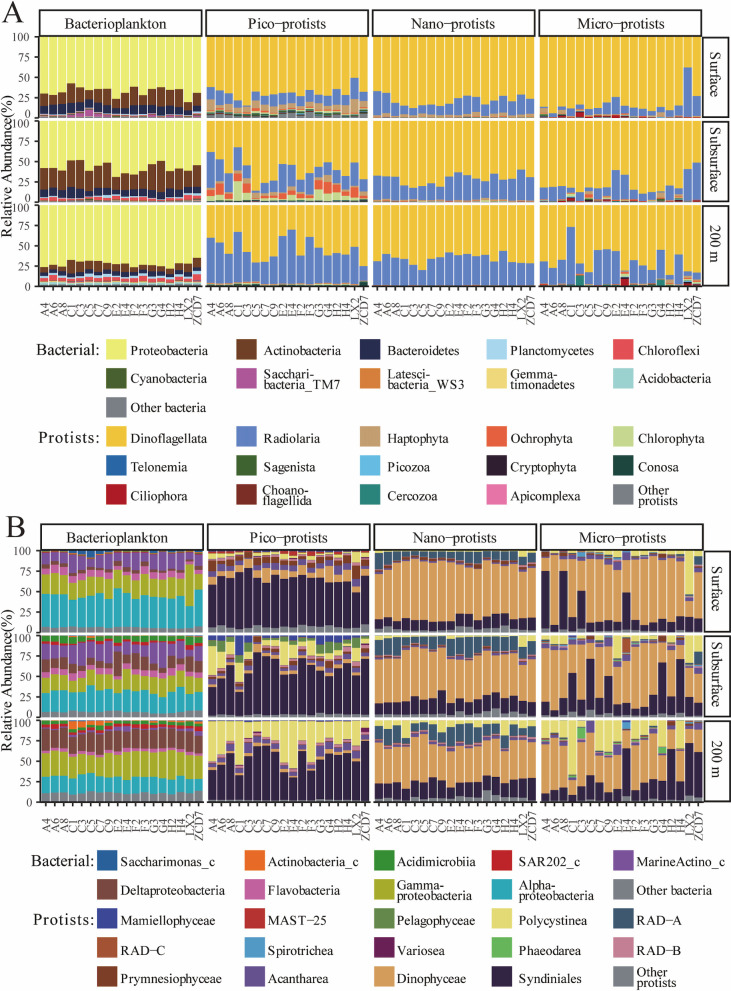
Community compositions of bacterioplankton and pico-, nano-, and micro-protists (**A**) at the phylum level (top 10 phyla and others) and (**B**) at the class level (relative abundance >5% in at least one of samples) across 54 samples for each size fraction.

Regarding the distribution patterns of all amplicon sequence variants (ASVs) in three water layers (Fig. S2), bacterioplankton and pico- and nano-protists displayed similar percentages of shared ASVs among three layers (relative sequence 85.3%–86.1%), while micro-protists had the lowest percentage of shared ASVs (relative sequence 66.3%). The community composition of bacterioplankton and three size fractions of protists exhibited different vertical distribution patterns (Fig. 2). Linear discriminant analysis (LDA) effect size (LEfSe) analysis helped to identify the differential taxa with the LDA value >4 in three water layers and thus reveal the dissimilarity of microbial communities (Fig. S3 to S6).

At the phylum level, very few taxonomic groups constituted more than 90% of the total microbial community composition data set. Specifically, in bacterioplankton communities, Proteobacteria (average relative abundance = 66%, including the classes Alpha-, Gamma-, and Deltaproteobacteria) was the most abundant phylum, followed by Actinobacteria (17%) and Bacteroidetes (8%). The class Alphaproteobacteria was significantly abundant in the surface layer and decreased with water depth (from 39% to 19%, LDA >4), while the class Deltaproteobacteria displayed the opposite (from 7% to 23%, LDA >4). The class MarineActino_c was most abundant in the subsurface layer (16%, LDA >4) and only accounted for 3% in the 200 m layer. Meanwhile, the class Gammaproteobacteria had the lowest relative abundance in the subsurface layer (19%) but was abundant in the 200 m layer (29%, LDA >4). Dinoflagellata and Radiolaria were the most dominant phyla in the protist communities. In pico-protist communities, Dinoflagellata mainly consisted of the class Syndiniales (55%, LDA <4), dominating in all the layers, while Radiolaria mainly consisted of Polycystinea, of which the average relative abundance increased from 7% to 31% with depths (LDA <4). Differently, Syndiniales (17%, LDA >4) and Polycystinea (4%, LDA >4) accounted for only minor proportions in nano-protist communities, which were dominated by Dinophyceae (55%, most abundance in surface layer, LDA >4) and RAD-A (14%, LDA <4) in all the layers. Compared to other groups, the compositions of micro-protist communities were more heterogeneous across different layers and among different samples from the same layer. Only a rare species, *Steenstrupiella steenstrupii* (total relative abundance of 0.3%), had the LDA score exceeding 3, which became the potential differential taxa in the surface layer. Dinophyceae (average relative abundance ± standard deviation = 50% ± 21%), Syndiniales (26% ± 19%), and Polycystinea (13% ± 15%) were still the dominant classes. In addition, the other abundant micro-protist phylum Cercozoa and classes including RAD-C, RAD-A, Acantharea, and Phaeodarea showed relative abundances of >10% in some samples.

**Fig 3:**
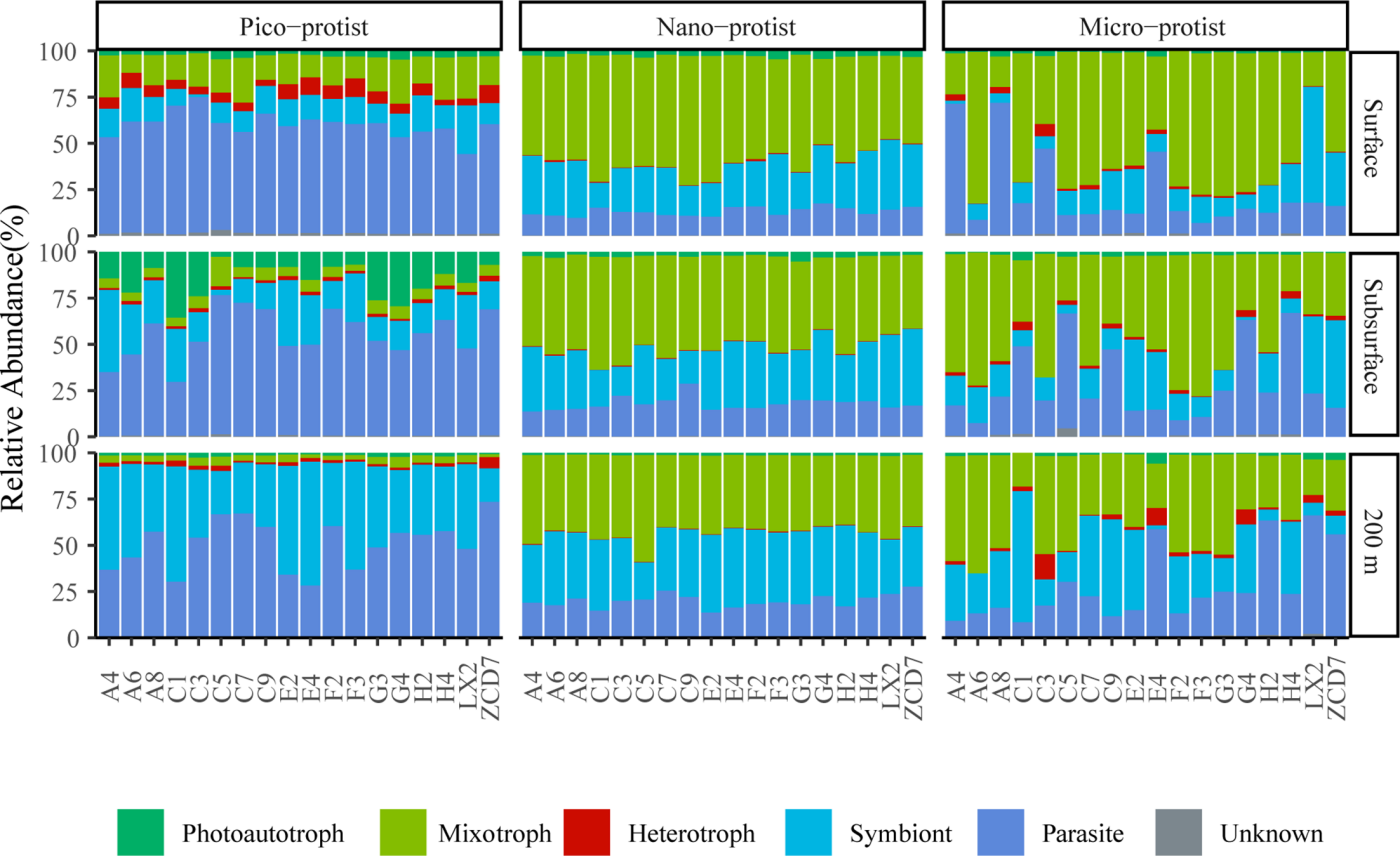
Community compositions of pico-, nano-, and micro-protists, grouped by trophic modes across 54 samples for each size fraction.

Furthermore, the compositions of trophic modes of protists of different sizes were analyzed by referring to previous literature and databases (Fig. 3, see Table S5 for trophic type assignments). More than 98% of protist ASVs were classified into trophic modes, accounting for a relative abundance of more than 99% of the total community composition. The highest relative abundance of symbionts (20% of ASVs) and parasites (55% of ASVs) was found in the pico-protist community, especially in the 200 m layer, where the sum of their average relative abundance exceeded 93%. The proportions of parasitic pico-protists decreased from 59% to 50% with depths, while symbiotic pico-protists increased from 13% to 42%. Heterotrophic pico-protists (5% of ASVs) mostly appeared in the surface layer (average relative abundance = 6%), while phototrophic pico-protists (4% of ASVs) showed the highest proportions in the subsurface layer (15%). In nano-protist communities, the proportions of mixotrophs (50%) were comparable with the sum of symbionts and parasites. The proportions of mixotrophs, symbionts, and parasites varied greatly among stations and layers in micro-protist communities.

### Alpha and beta diversity of microbial communities

The Shannon’s diversity index, Simpson’s index, and ASV richness were used to evaluate the α-diversity of the microbial community (Fig. S7). In general, pico-protists had the highest Shannon’s diversity index and ASV richness index (*P* < 0.05). The three indices of bacterioplankton and nano-protist communities showed similar increasing trends with increasing water depth, while indices of pico-protist communities were the highest in the surface layer. All indices of the micro-protist community differed insignificantly among water layers.

**Fig 4:**
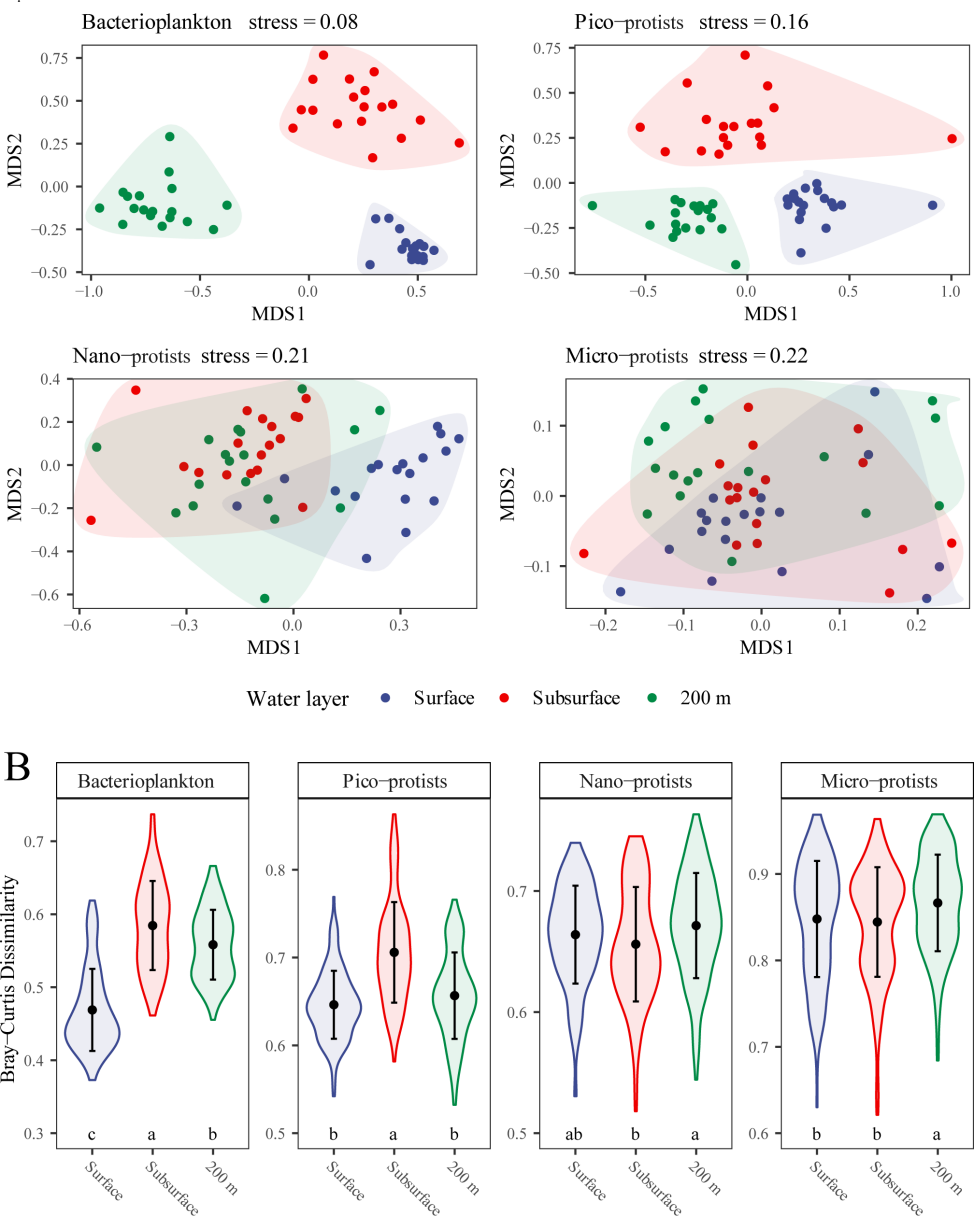
(**A**) NMDS ordination of microbial communities in three water layers. (**B**) Violin plots of Bray-Curtis dissimilarity of microbial communities in each water layer. Black points and error bars represent the mean values and standard deviations, respectively. Letters indicate significant differences (*P* < 0.05) among water layers calculated by LSD tests.

The analyses of nonmetric multidimensional scaling (NMDS), violin plots, and permutation multivariate analysis of variance (PERMANOVA) showed the differences in the β-diversity of microbial communities among three water layers, and the disparities between water layers diminished as cell size increased (Fig. 4; Table S2). Specifically, the compositions of bacterial communities were profoundly different among all water layers, with obvious separation in NMDS (stress = 0.08), large explanations of difference in PERMANOVA (*R*^2^ >0.3 and *P*_adjusted_ = 0.001), and significant differences of Bray-Curtis dissimilarity by least significant difference (LSD) tests (*P* < 0.05). For the protist communities, the distinction among communities of different water layers becomes less obvious as cell size increased, likely attributable to a greater degree of overlap in community composition (Fig. 4A), as evidenced by higher adjusted *P*-values from PERMANOVA (*P*_adjusted_ >0.01 for micro-protist communities). Additionally, we observed higher Bray-Curtis dissimilarities among stations within each water layer for nano- and micro-protist communities compared to pico-protist communities. This suggests that larger-sized protist communities (nano- and micro-protists) exhibit greater intra-group variability within the same water layer than their smaller-sized counterparts (pico-protists) (Fig. 4B).

### Environmental drivers of microbial communities

**Fig 5:**
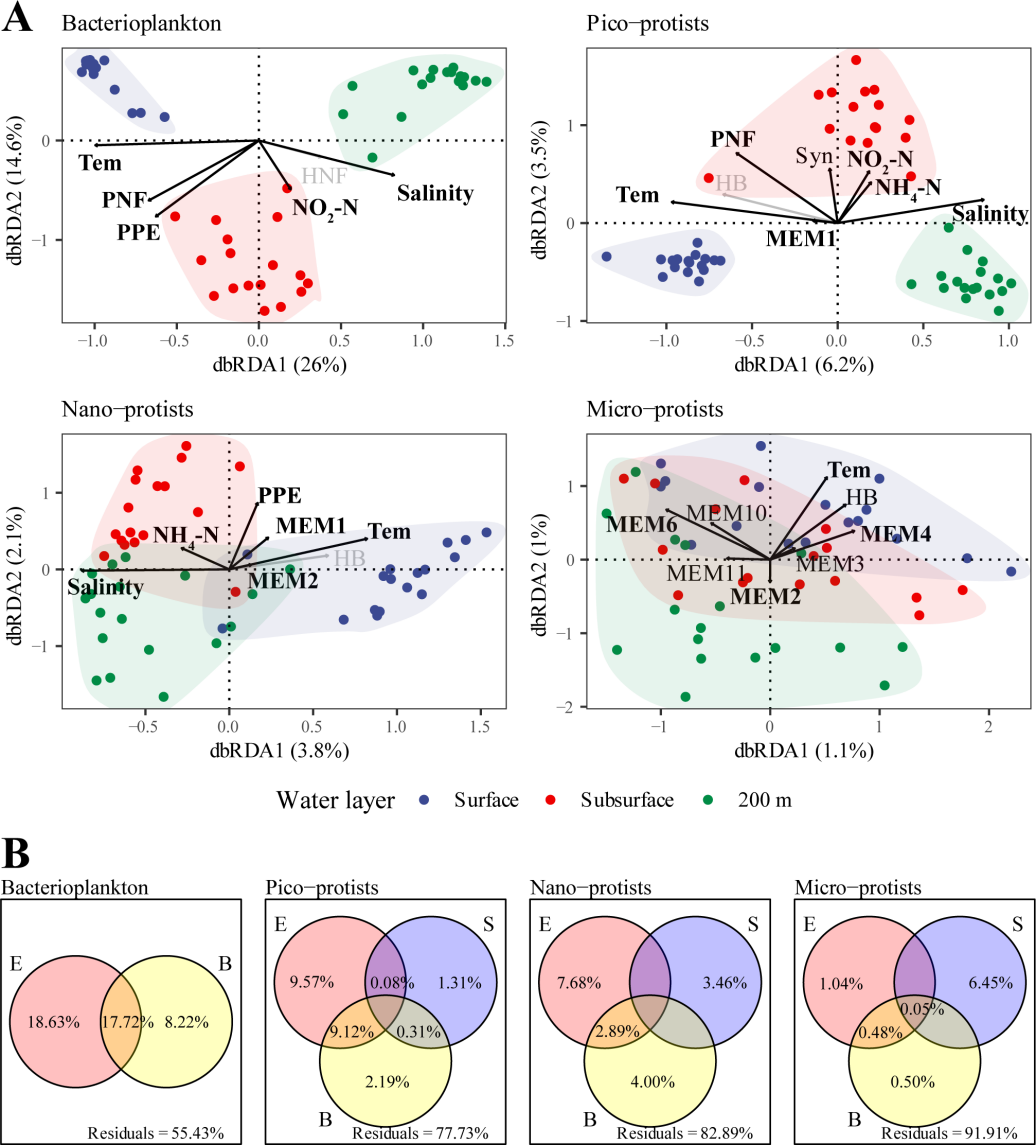
The variations of bacterioplankton and pico-, nano-, and micro-protist communities are explained by spatial, environmental abiotic, and biotic variables, respectively. (**A**) RDAs show the microbial communities’ compositions in relation to non-significant (*P* > 0.1, gray fonts) and significant (*P* < 0.1, black fonts; *P* < 0.05, bold fonts) variables. (**B**) VPAs show the contributions of spatial factors (S), environmental abiotic factors (E), and biotic factors (B) on the compositions of the microbial communities, values <0 are not shown. The abbreviations of environmental and spatial variables are Tem, temperature; NO_2_-N, nitrite; NH_4_-N, ammonium; PO_4_-P, phosphate; Syn*, Synechococcus*; HB, heterotrophic bacteria; PPE, photosynthetic picoeukaryotes; HNFs, heterotrophic nano-sized flagellates; PNFs, pigmented nano-sized flagellates; MEM1, 2, 3, 4, 6, 10, and 11, a set of spatial factors which were generated by distance-based Moran’s eigenvector maps. Note: Only variables with variance inflation factors less than 10 and significant in the forward model choice based on *R*^2^ and *P*-values were retained in the analysis.

The results of distance-decay patterns showed no significant correlations between the Bray-Curtis dissimilarities of four groups of microbial communities and geographic distances among stations within every water layer (Fig. S8). Distance-based redundancy analysis (dbRDA) and variation partitioning analysis (VPA) were performed to determine the effects of environmental factors (only abiotic factors), biotic factors, and spatial variables on microbial communities’ structure (Fig. 5). Basically, the environmental factors played more important roles in shaping bacterioplankton community compared to all the protist communities, especially the abiotic factors, while the spatial factors explained more variations in protist communities than in bacterial communities (Fig. 5B). For the bacterioplankton community, the environmental abiotic factors uniquely explained a substantial portion of the community variation (18.63%), markedly exceeding the contribution of unique biotic factors (8.22%). For the pico-protist community, the uniquely environmental abiotic factors (9.57%) contributed more than the biotic factors (2.19%) and the spatial factors (1.31%). The importance of spatial factors exceeded the environmental abiotic and biotic factors in both the nano- and micro-protist communities. In addition, the unexplained proportion remained the lowest in the bacterial community (55.43%) and increased progressively with cell size, reaching up to 91.91% in the micro-protist community (Fig. 5B). The cumulative variance explained by the first two dbRDA axes also showed a declining trend as cell size increased (Fig. 5A).

Among the environmental factors, salinity and temperature were the two most important factors explaining the variability of bacterial and pico- and nano-protist communities in the surface and 200 m layer, respectively (Fig. 5A; Fig. S9 to S11). The nutrients (i.e., NO_2_-N and NH_4_-N) and biotic factors had strong positive effects on communities and taxa in the subsurface layer (Fig. 5A; Fig. S9 to S11).

### Niche breadth and microbial community assembly processes

**Fig 6:**
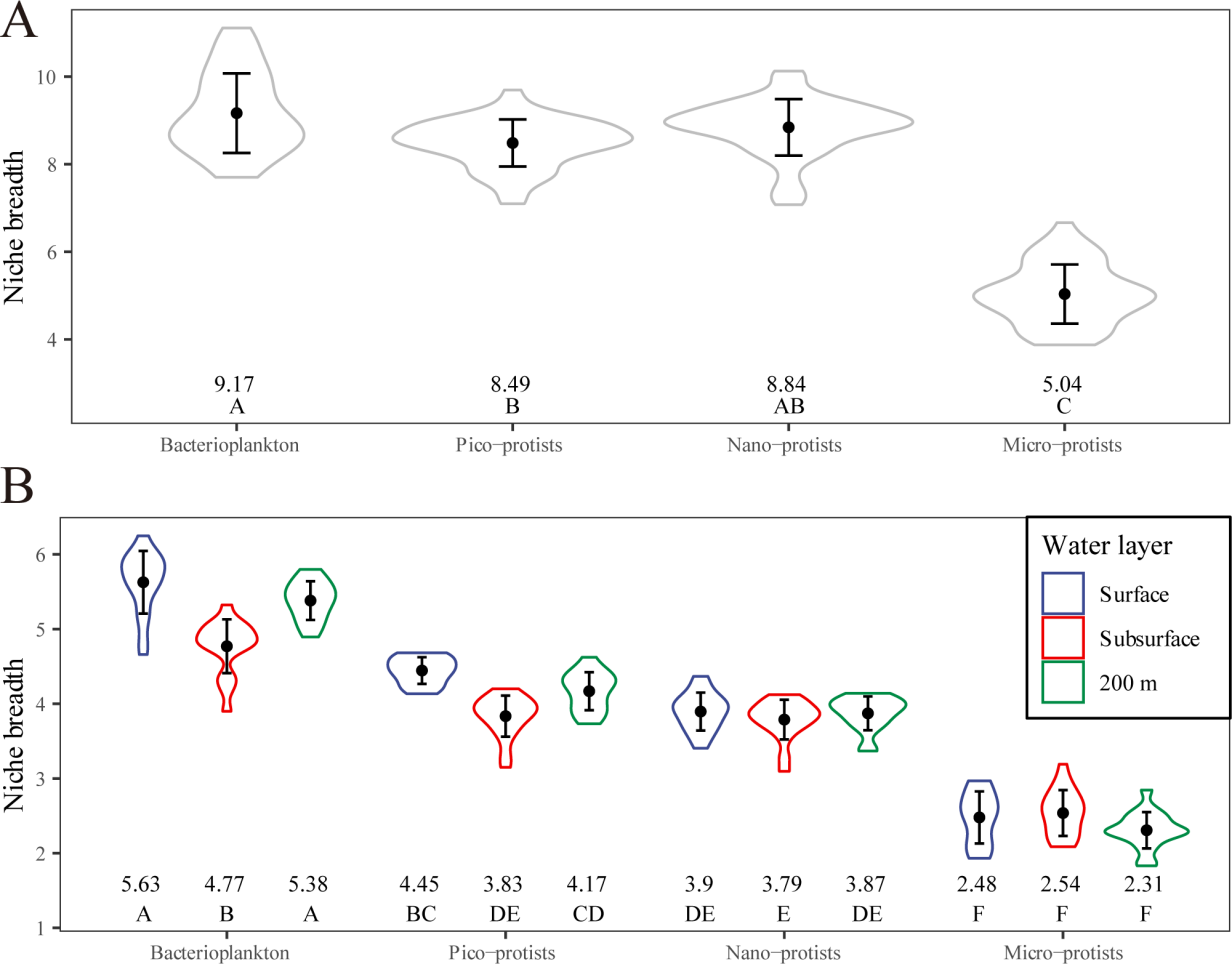
Boxplots of community-level niche breadth of bacterial and three size fractions of protist communities (**A**) across all depths and (**B**) in each water layer. LSD tests are calculated among groups. The numbers below the box indicate the mean values. Letters indicate significant differences (*P* < 0.05).

There were significant differences in community-level niche breadth among the four size fraction communities (Fig. 6). For all the samples (Fig. 6A), bacterioplankton communities had the highest average niche breadth (9.17), while micro-protist communities had the lowest (5.04). The niche breadth of nano-protist communities (8.84) was slightly higher than that of pico-protist communities (8.49). Comparison by water layer revealed that the community-level niche breadth decreased with increasing microbial size fraction (Fig. 6B), from 5.63 for the bacterial community in the surface layer to 2.31 for the micro-protists in the 200 m layer. In each water layer, the community-level niche breadth of microbial communities was highest in the surface layer, followed by the 200 m water layer, and lowest in the subsurface layer, except for micro-protist communities, which showed the highest community-level niche breadth in the subsurface community.

**Fig 7:**
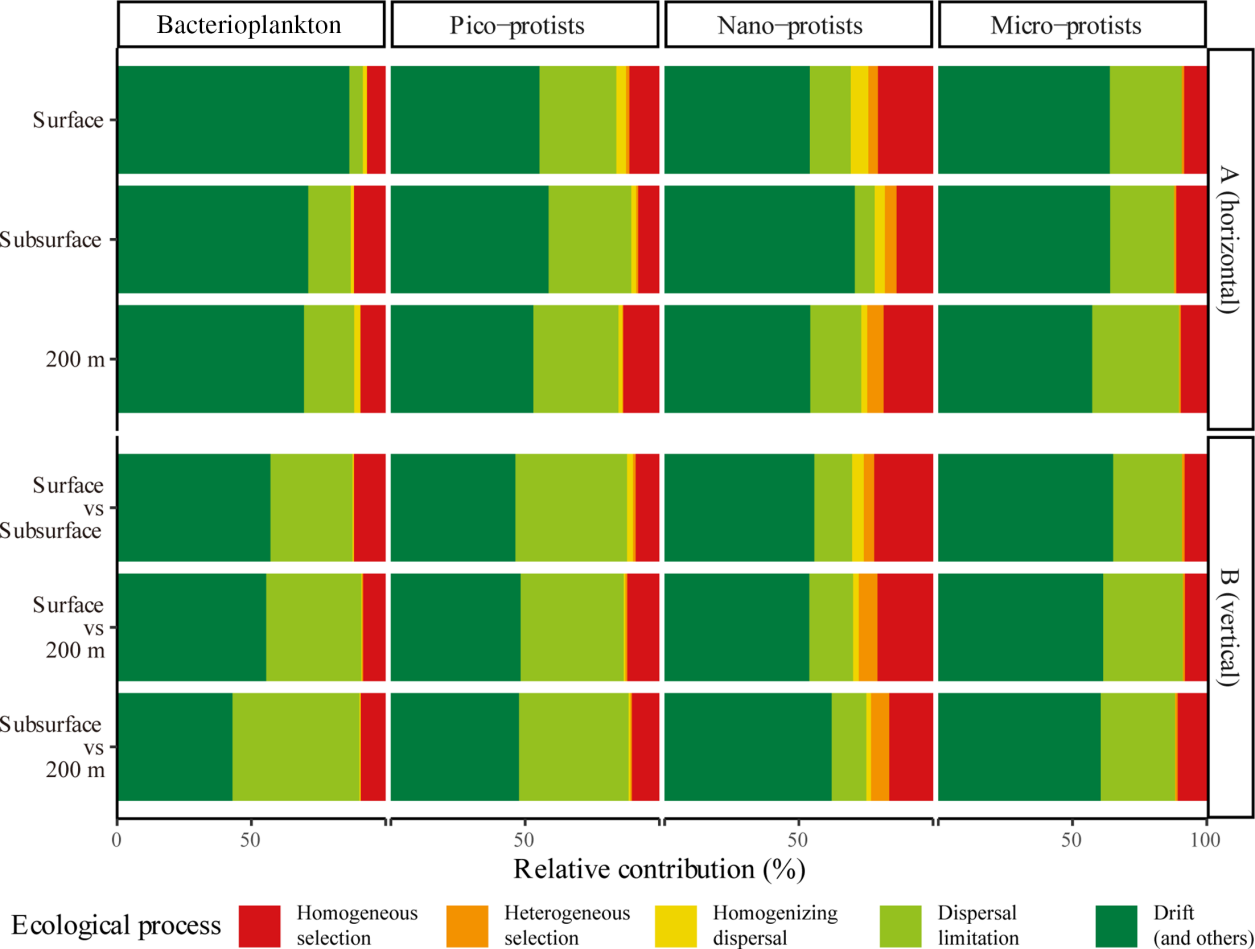
Relative contribution of different processes for microbial assembly at the (**A**) horizontal scale and (**B**) vertical scale based on phylogenetic bin-based null model analysis (iCAMP). Drift (and others) includes drift, diversification, weak selection, and/or weak dispersal.

The results based on phylogenetic bin-based null model analysis (infer community assembly mechanisms by phylogenetic-bin-based null model analysis [iCAMP]) suggested that stochastic processes, consisting of homogenizing dispersal, dispersal limitation, drift, and others (including diversification, weak selection, and/or weak dispersal), dominate community assembly (Fig. 7). In particular, drift was the dominant process in shaping four size fraction communities, ranging from 53.0 to 86.5% in horizontal scale, and 42.0% to 65.1% in vertical scale. Dispersal limitation also played an essential role in community assemblages, with contributions ranging from 5.0% to 32.3% in horizontal scale, and 12.9% to 47.2% in vertical scale. In contrast, homogeneous selection, heterogeneous selection, and homogenizing dispersal accounted for only a small fraction of the variation in the microbial community, with relative contributions ranging from 6.9% to 22.0%, 0.01% to 7.0%, and 0.03% to 6.6%, respectively. Compared to larger protists, bacterioplankton were very limited in vertical dispersal and increased with depth, with Cohen’s d ranging from −27.6 to 9.0 and *P* < 0.05 (Fig. 7; Table S3). Dispersal limitation also accounted for a considerable proportion of the assemblage of the pico-protist community, but the differences were small between water layers, with Cohen’s d ranging from −7.8 to 1.0. Deterministic processes (homogeneous selection and heterogeneous selection) accounted for the highest proportion of nano-protist community assembly, with relative contribution from 18.0% to 27.7% (Fig. 7). Micro-protist community assembly displayed homogeneity, with only one group having *P* < 0.05 in homogeneous selection.

Based on the iCAMP, the ASVs of bacterioplankton and pico-, nano-, and micro-protists were classified into 153, 184, 83, and 122 phylogenetic bins, respectively (Fig. S12 to S16). The results suggested that drift dominated the vast majority of microbial bins (ranging from 54.2% to 91.8% of bin numbers), followed by dispersal limitation (ranging from 6.0% to 40.0% of bin numbers). This approach further revealed that some taxa had different causes of formation, despite having similar vertical distribution characteristics. For example, some of the ASVs of the class Alphaproteobacteria were dispersal limited, and some of the ASVs of the class MarineActino_c were dominated by drift, contributing to a decrease in the relative abundance of these two classes of bacteria with increasing water depth (Fig. 2A; Fig. S12). Additionally, different sizes of the same protists’ class were not consistently governed by similar ecological processes. Pico-sized Acantharea was dominated by homogeneous selection (Fig. S13), while micro-sized Acantharea was dominated by drift (Fig. S15). Even nano-sized Dinophyceae were partly dominated by homogeneous selection and almost exclusively by drift processes (Fig. S14). Although the relative contribution of deterministic processes (homogeneous selection and heterogeneous selection) was small, when focusing on bins with a single trophic mode dominated by deterministic processes, it was found that these bins were composed of pico- and nano-sized mixotroph (Fig. S16).

## DISCUSSION

### The diversity and trophic mode composition of microbial communities were associated with size fraction

Data regarding protists of varying sizes are lacking, and our study offers new insights into size-fractionated protistan community composition and ecological functions. Comparable to the global survey of the *Tara* Oceans project (30), protist taxonomic diversity was higher in smaller organisms and decreased with increasing organism size fractions (Fig. S7A). The specific community composition of each size fraction corresponds to the cell size, while the great distinction in protists of different cell size fractions refers to the ecological function.

Parasitic taxa had extremely high proportions in pico-protist communities, especially at the surface layer, and were lower in nano- and micro-protist communities (Fig. 3), which may be related to the life cycles of the parasitic taxa. Numerous pico-parasites are free-living spores that spread widely in the ocean with the help of water currents (30). Typical and abundant parasitic taxa such as Syndiniales kill their hosts, including protists and metazoans, and subsequently release free-living dinospores (31). This life cycle strategy contributes to their widespread global distribution and even dominance in polar waters (31, 32). As considered to be an advantageous strategy in marine ecosystems (33), parasitic taxa contribute nearly half of the pico-protist ASV library (30) and have been proposed to strongly influence microbial food webs and community structure (34, 35).

In addition to the importance of pico-parasitic taxa in the microbial food web, our findings also revealed dominant mixotrophic and symbiotic nano- and micro-protists in the inorganic nutrient-poor area of the SCS. The mixotrophic mode has been found in a variety of algal lineages, including Dinoflagellata, Haptophyta, and Chlorophyta. Most Dinophyceae were identified as mixotrophic organisms, usually with cell sizes >5 µm (36, 37), which were more abundant in the nano- and micro-protist communities. As previously found by Unrein et al. (38), most of the mixotrophic taxa in the <20 µm fraction belong to nano-protists (Fig. 3). A few pico-protists also feed on bacterioplankton, such as Prymnesiophyceae (belonging to Haptophyta) (39). In nutrient-limited environments, mixotrophic microorganisms are capable of supplementing their nutrient requirements through grazing and are therefore more competitive than heterotrophic microorganisms (40). In addition to this, mixotrophic protists contribute significantly to bacterivory by accounting for approximately half of bacterial consumption, thereby facilitating the transfer of biomass to higher trophic levels and enhancing the efficiency of oceanic carbon storage (41, 42). The results of significant Spearman correlations and non-negligible biotic explanations in VPA have suggested that strong biotic interactions (e.g., predation) were prevalent between these protists and bacterioplankton, especially in the subsurface layer (Fig. 5; Fig. S9 to S11). Dilution experiments had similarly revealed that flagellate predation exerted significant control over the abundance and structure of bacterial communities in the South China Sea (43). Comparable findings had been documented in investigations conducted across other marine regions (44–46).

### Environmental factors regulated the vertical distribution of the microbial community

Our results showed that in the epipelagic zone of SCS, the smaller the cells, the more pronounced the stratification of the microbial community (Fig. 4). Although microbial community assembly was dominated by stochastic processes (Fig. 7), resulting in a low proportion of dbRDA explainable variation (Fig. 5A) (27), it was still worthwhile to consider the effects of multiple environmental factors on the vertical structure of microbial communities. Of the environmental factors we measured, temperature and salinity were suggested as the important abiotic factors impacting microbial communities in the surface and 200 m layers (Fig. 5A). As a key factor controlling the metabolic reaction rates and pathways of microorganisms (47), temperature has been reported by numerous studies that showed strong correlations with the diversity of the marine microbial community and species composition, especially in the epipelagic zone (48, 49). Although the observed salinity variation in this study was relatively small (~1 unit; Table S1), the results of the Mantel and partial Mantel tests suggested that temperature and salinity exerted independent effects on microbial community structure beyond the effects of vertical scale (Table S4). Therefore, even subtle changes in salinity, when coupled with other factors such as temperature, nutrients, and light, may contribute to microbial community differentiation in the water column (7, 50).

Unlike the surface and 200 m layers, the microbial community structure in the subsurface layer was mainly influenced by nutrients (NO_2_-N and NH_4_-N) and the biotic factors (e.g., the abundance of PNF, PPE, and Syn) (Fig. 5A). Subsurface layer was nutrient richer and had higher abundance of photosynthetic organisms (Fig. 1C). Previous studies have revealed that nutrient supply (especially the addition of nitrogen and phosphorus) significantly promoted phytoplankton growth and biomass increase, even exceeding the effects of temperature changes (51, 52). In oligotrophic ecosystems, nutrients are important limiting factors for phytoplankton growth (15, 53, 54). Naturally, different phytoplankton communities vary in their environmental adaptability and respond differently to changes in nutrient conditions (51). For example, *Prochlorococcus* is better adapted to oligotrophic environments but tends to be replaced by other larger, faster-growing species when nutrient conditions improve (55). Microbial taxa could also coordinate their metabolism through diel cycles and exhibit synergistic metabolic patterns, enabling communities to more efficiently utilize limited resources in oligotrophic environments (56). Furthermore, predatory, parasitic, and symbiotic relationships between planktonic microorganisms influence microbial community structure and nutrient cycling (34, 57). In this study, insufficiently detailed analyses of biotic relationships such as selective grazing and viral lysis may obscure our ability to explain community variation (58, 59), especially for larger organisms (Fig. 5).

### Stochastic processes dominated microbial community assembly

The extent to which and how various ecological processes affect the distribution of marine microorganisms is a controversial issue (60). In different ecosystems, the assembly processes between bacteria and protists or among different size fractions can range from highly similar to vastly dissimilar (16, 17, 61–63). Our results indicated that stochastic processes play a dominant role in the assembly of four size fraction communities, without exactly being consistent with either the “size-plasticity” or “size-dispersal” hypotheses. On the one hand, as suggested by the “size-plasticity” hypothesis, community-level niche breadth decreased with increasing cell size (Fig. 6), but the relative importance of deterministic processes did not increase as a consequence (Fig. 7). On the other hand, not as expected from the “size-dispersal” hypothesis, both pico-sized cells (bacteria and pico-protists) and larger micro-protists were dispersal limited, whereas nano-protists with intermediate cell sizes were least affected by dispersal limitation (Fig. 7).

This result was partly expected, as environmental and spatial factors explain low community variation (Fig. 5), and the absence of distance-decay patterns in both bacterial and protist communities implied the importance of stochastic processes (Fig. S8). Additionally, it has been suggested that there was a significant positive correlation between local abundance and community dispersal scales for microorganisms (28). For pico-sized cells, their high cellular abundance and short generation time were expected to result in more thorough distribution across the water column, given the stochastic nature of dispersal (8, 64). Furthermore, this high passive dispersal ability in association with water stratification of the surface and subsurface water currents in the studied area during the summer allowed for a greater relative contribution of dispersal limitation at vertical scales (Fig. 1B) (26). This was also valid for larger micro-protists, which typically have longer generation times and smaller population densities, and are therefore more sensitive to local extinctions and ecological drift (28). Coupled with the lower community-level niche breadth (Fig. 6), which suggested a weaker adaptation to environmental changes (65), larger size fraction organisms usually possess lower α-diversity (Fig. S7B) and were dominated by drift (Fig. 7).

Our findings highlighted the distinct ecological characteristics of nano-protists in microbial communities. Compared with other microorganisms, nano-protists experienced greater homogenizing selection and were subject to less dispersal limitation. A study on lake ecosystems has also found that pico/nano eukaryotes (0.22 µm–20 µm) assemble independently from bacteria and micro-eukaryotic (20 µm–200 µm) communities (63). As iCAMP revealed the uniqueness of nano-mixotrophic protists dominated by homogeneous selection, this was probably related to the high relative abundance of mixotrophic nano-protists. This highlighted the importance of reconsidering the role of nano-protists, especially nano-mixotrophic protists, in the microbial food web (66).

### Possible responses of marine microorganisms to future environmental changes

Bacteria and unicellular protists are critical components of the microbial food web and are central to biogeochemical cycles (67). In this study, we observed that although the community was dominated by stochastic processes, deterministic processes affected nano-protists more than other components (Fig. 7). Additionally, community-level niche breadth decreases with increasing cell size (Fig. 6), suggesting a differential impact of environmental filtering on four groups of microorganisms (65). Given this, the abundance and community structure of bacteria and protists may change unequally, which would result in changing the strength of consumer predation and parasitic interactions between microorganisms (16, 68), and even trophic mismatch, with serious ecological consequences (69, 70) under future scenarios of changes in the marine environment. Particular attention needs to be given to the more deterministic process-driven mixotrophic protists, which are not only involved in primary production but also consume bacteria and play a crucial role in the structure of microbial food webs (41, 42). Although this was theoretical speculation based on the comparison of marine microbial community assembly mechanisms in bacterioplankton and three size fractions of protists, it was supported by recent biogeochemical studies (71–73). Therefore, in the future, we need to analyze the impacts of climate change on marine microorganisms and microbial food webs from a more detailed and systematic perspective.

## MATERIALS AND METHODS

### Study location and sampling

The SCS is the world’s second-largest marginal sea and is an oligotrophic region with nitrogen deficiency (74). It has a tropical oceanic monsoon climate with high temperature and humidity all year round. Affected by the monsoon, there are mainly two seasons: summer and winter. Our sampling of 18 stations was conducted in the summer (21 August to 22 September 2021) (Fig. 1A), from three water depths in the euphotic zone: the surface layer (5 m), the subsurface layer (90 m at most stations, 75 m–87 m at C-series stations), and the bottom layer of the euphotic zone (200 m). An SBE9 CTD (Seabird Electronics Inc., USA) was used to collect seawater and measure temperature, salinity, dissolved oxygen, and density *in situ*. The concentrations of NO_3_-N, NO_2_-N, PO_4_-P, and SiO_4_-Si were analyzed with an AA3 continuous flow analyzer (SEAL Analytical Inc). NH_4_-N was determined by sodium hypobromite oxidation spectrophotometry. Pro, Syn, HB, and PPE were fixed with a final concentration of 0.5% (vol/vol) glutaraldehyde, and their abundance was run with a FACSAria flow cytometer (Becton Dickinson, USA) equipped with laser emitting at 488 nm (75). Following previous protocols (76), nano-sized flagellates (NFs) were also fixed with a final concentration of 0.5% (vol/vol) glutaraldehyde, then stained with 4′6-diamidino-2-phenylindole and filtered onto 0.8 µm pore size black nucleopore filters (25 mm in diameter; Millipore, MA, USA) at low pressure (<100 mm Hg). Filters were mounted to glass slides and stored at −20°C in the dark until observed by epifluorescence microscope (Leica Microsystems, Germany). Identification of HNFs and PNFs was based on blue fluorescence of nucleic acid under UV excitation and red or orange autofluorescence of chlorophyll under blue excitation. At least 50 random views were chosen for each sample to obtain reliable estimates of NF abundance. For microbial community analysis, 10 L of seawater was pre-filtered through a 200 µm filter and then sequentially filtered through 20 µm, 2 µm, and 0.2 µm filter membranes (47 mm diameter, Millipore, Billerica, MA, USA), obtaining bacterioplankton (0.2 µm–2 µm) samples and three size fractions of protist filter samples, i.e., pico-protist (0.2 µm–2 µm), nano-protist (2 µm–20 µm), and micro-protist (20 µm–200 µm). Filters were stored at −80°C until DNA extraction.

### DNA extraction and PCR

DNA was extracted using a DNeasy PowerWater kit (Qiagen, USA) following the manufacturer’s instructions. For bacterioplankton, the V5-V6 region of the 16S rRNA gene was amplified with primers 787F (5′-ATTAGATACCCNGGTAG-3′) and 1046R (5′-CGACAGCCATGCANCACCT-3′) (77). A quintuple repetition of each sample was amplified as follows: 94°C for 3 min, 25 cycles of 94°C for 30 s, 55°C for 45 s, and 72°C for 1 min, and a final extension at 72°C for 5 min. For protists, the V4 region of the 18S rRNA gene was amplified with primers 3NDF (5′- CAAGTCTGGTGCCAG-3′) and V4_euk_R2 (5′-ACGGTATCTRATCRTCTTCG-3′) (78). A quintuple repetition of each sample was amplified as follows: 95°C for 2 min, 35 cycles of 95°C for 30 s, 55°C for 30 s, and 72°C for 45 s, and a final extension at 72°C for 10 min. Forward and reverse primers were tagged with 2 bp links and 8 bp barcodes to allow the pooling of multiple samples in one run of sequencing and later differentiation of different samples (79). The PCR products were purified using AMPure XP beads (Beckman Coulter, USA) and quantified by Nano-300 (Allsheng, China). Samples belonging to four microbial groups were respectively mixed in equimolar concentrations to construct four individual amplicon libraries for sequencing by a commercial sequencing company (Novogene Corp., Ltd., Beijing, China) using Illumina Hiseq 2500 platforms with PE250 strategy (Illumina, USA) according to standard protocols.

### Sequencing data analyses

Sequencing data processing was performed on Mothur v.1.47.0 (79) following MiSeq standard operating procedure (http://mothur.org/wiki/miseq_sop/) with the steps of sequencing data quality control, ASV clustering, and species classification. Specifically, read pairs were aligned, and the tags and primers of reads were removed. To reduce sequencing and PCR error, reads with only one or two sequences were removed. Chimeras were detected using the UCHIME algorithm (80), and if there were flagged chimeras, they were removed from all samples. The remaining high-quality reads with suitable lengths (400 bp–460 bp for protists and 200 bp–250 bp for bacteria) were clustered to distinguish ASVs. The SILVA nr v.138 database (81) and PR^2^ protist v.4.14 database (82) were used for bacterial and protist taxonomic assignment, respectively. To avoid distortion of the relative abundance of DNA sequences of microbes, non-bacterial or non-protistan sequences (e.g., “unknown”, Archaea, Nucleomorphs, Fungi, Streptophyta, and Metazoa) were removed. Lastly, 216 samples from 18 stations were rarefied to the lowest sequence count (7,000 sequences for bacterioplankton samples, 5,000 sequences for both pico- and nano-sized protist samples, and 400 sequences for micro-sized protist samples) from each data set. Thus, a total of 8,341 bacterioplankton ASVs, 13,753 pico-protist ASVs, 13,158 nano-protist ASVs, and 3,026 micro-protist ASVs were retained.

Referring to previous literature reports (83, 84), the functional groups of protist ASVs were annotated into photoautotroph, symbiont, parasites, heterotroph (mainly phagotroph), and mixotroph, where mixotroph refers to photosynthetic species that also ingest food by phagocytosis or osmotrophy, and symbiont refers to heterotrophic species that retain prey plastids or symbionts (85) (Table S5).

### Statistical analyses

All statistical analyses were performed in R v.4.2.1 software (86), visualized using the “ggplot2” R package. Considering the significant differences in sequencing depth for microbial communities of different size fractions, we used a combination of rarefaction and coverage-based methods. For α-diversity analysis, we used a coverage-based diversity estimation method to avoid discarding valuable data (87). For other analyses, we applied rarefaction to normalize the sequencing depth across samples, ensuring a consistent comparison of community composition.

α-Diversity including ASV richness, Shannon’s diversity, and Simpson’s diversity was calculated at fixed coverage (90.5%) using unrarefied data and completed with the "iNEXT" R package. Fixed coverage was determined by the average coverage of bacterial and protist samples. Differences in α-diversity among water layers were analyzed with LSD test, using the “agricolae” R package. For community composition, the “microeco” R package was used to create Venn diagrams to interpret shared or special taxa in the three water layers. Differences of taxa among water layers were analyzed by the LEfSe algorithm in the “microeco” R package. Only lineages with LDA values >4 were displayed as the differential taxa in each water layer.

Prior to multivariate statistical analyses, the ASV tables were Hellinger-transformed, and the environmental variables were log(x + 1) transformed. The following analysis was carried out using the “vegan” R package. Principal component analysis was conducted to test the explanatory power of environmental variables on the variation of samples. A set of spatial factors was generated by distance-based Moran’s eigenvector maps (which was called PCNM in early papers [88]) based on the longitude and latitude coordinate of each sampling station using the “adespatial” R package. The β-diversity was calculated on the Bray-Curtis dissimilarity metric and visualized with NMDS. The significant differences in microbial communities among water layers were tested by PERMANOVA using the Bray-Curtis dissimilarity metric. To explore the effects of biotic, abiotic, and spatial factors on microbial communities, dbRDA and VPA were performed. The environmental variables with variance inflation factors >10 and insignificant in the forward model choice based on *R*^2^ and *P*-values were filtered in advance. Distance-decay curves were plotted by linear regression of the Bray-Curtis dissimilarity of microbial communities against the pair-wise geographical distances matrix based on longitudinal and latitudinal coordinates of each sampling site in each water layer. In addition, correlation analyses between the differential taxa with LDA value >4 and the environmental parameters were tested using the Spearman rank correlation coefficient. It is important to note that, in order to reveal the biotic interactions between different size groups, the biotic factors considered for each size group did not include the abundance data of organisms within the same size group. Additionally, Mantel and partial Mantel tests were conducted to evaluate the correlations among temperature, salinity, and depth and their relationships with microbial community composition. Bray-Curtis dissimilarity matrices were applied to represent community composition, while Euclidean distance matrices were used for environmental variables.

The assessment of the environmental adaptation of microbial communities was carried out using an index of community-level niche breadth, which was the average of the Levins’ niche breadth index for all taxa belonging to a single community (16, 89). Levins’ niche breadth index (B) was calculated following the description of Pandit et al. (89) using the “spaa” R package:


$$B_{j}=1/\sum_{i=1}^{N}P_{ij}^{2}$$

where *B_j_* represents the habitat niche breadth of ASV *j* in a metacommunity; *N* is the total number of communities in the metacommunity; and *P_ij_* is the proportion of ASV *j* in resource state *i,* i.e., the abundance of ASV *j* in community *i* divided by the abundance of ASV *j* in the metacommunity. The *B* value ranges from [1, N], and the higher value indicates that the ASV *j* is widely and evenly distributed in the metacommunity. Community-level niche breadth was calculated for all depths and each water layer, respectively, and metacommunities were defined as the set of communities at all depths and the set of all communities in each water layer, respectively. Differences in community-level niche breadth among communities were analyzed with the LSD test.

### Null module analysis

Decoupling between taxonomy and function can occur in marine microorganisms (90), requiring more careful consideration of the role of phylogenetic relationships in community assembly. iCAMP provided a robust framework for analyzing microbial community assembly by categorizing taxa into phylogenetic bins and assessing ecological processes through null model analyses (91). It offered higher precision, sensitivity, and accuracy in quantifying ecological processes compared to previous approaches (91). We therefore quantified community assembly mechanisms using iCAMP with the “iCAMP” R package. In brief, the analysis involved the following steps (91): the observed taxa were first divided into groups (“bins”) based on phylogenetic relationships. The β net relatedness index (βNRI) was calculated separately for each bin based on 1,000 times randomization null module analyses of phylogenetic diversity. The modified Raup-Crick metric (RC) was obtained based on taxonomic β-diversity. The βNRI was used to determine if the assembly of a bin was stochastic (|βNRI| ≤ 1.96) or deterministic (|βNRI| > 1.96), and the RC was used to identify the specific stochastic process. Specifically, for each bin, the fraction of pairwise comparisons with βNRI < −1.96 was considered as homogeneous selection, βNRI > 1.96 was considered as heterogeneous selection, |βNRI| ≤ 1.96 and RC <−0.95 was considered as homogenizing dispersal, |βNRI| ≤ 1.96 and RC >0.95 was considered as dispersal limitation, and |βNRI| ≤ 1.96 and |RC| ≤ 0.95 was considered as drift and others (including diversification, weak selection, and/or weak dispersal). Thereafter, the fractions of individual processes across all bins were weighted according to the relative abundance of each bin and aggregated to estimate the overall relative importance of individual processes at the community level. Finally, bootstrapping was used to estimate the variation of relative importance of each process in each water layer and compare the difference between them.

## Data Availability

The raw sequence data have been deposited in the NCBI Sequence Read Archive under the BioProject accession number PRJNA1023690.

## SUPPLEMENTAL MATERIAL

Supplemental material aem.00436-25-s0001.docx Figures S1 to S16; Tables S1 to S4.

Table S5 aem.00436-25-s0002.xlsx Trophic types of different taxonomic groups of microbial protists.

ASM does not own the copyrights to Supplemental Material that may be linked to, or accessed through, an article. The authors have granted ASM a non-exclusive, world-wide license to publish the Supplemental Material files. Please contact the corresponding author directly for reuse.
