# Supplementary material for: Contrasting patterns in diversity and community assembly of bacterioplankton and three size fractions of protists in the South China Sea
Source: Appl Environ Microbiol. 2025 Jun 26;91(7):e00436-25. doi: 10.1128/aem.00436-25 (PMC12285259; doi:10.1128/aem.00436-25)
Supplement: Supplemental material — Figures S1 to S16; Tables S1 to S4. [file aem.00436-25-s0001.docx]

**Supplementary Information**

**Contrasting patterns in diversity and community assembly of bacterioplankton and three size fractions of protists in the South China Sea**

Author information

Xinyi Zheng ^1, †^, Xin Guo ^2,3, †^, Xiaoqing Lin^1^, Lingfeng Huang^1 *^

^1^ Key Laboratory of the Ministry of Education for Coastal and Wetland Ecosystems, College of the Environment and Ecology, Xiamen University, Xiamen, China

^2^State Key Laboratory of Marine Environmental Science, College of Ocean and Earth Sciences, Fujian Key Laboratory of Marine Carbon Sequestration, Xiamen University, Xiamen, China

^3^ Carbon Neutral Innovation Research Center, Xiamen University, Xiamen, China

^†^Should be considered the joint first author.

^*^Corresponding authors: Lingfeng Huang. E-mail: [huanglf@xmu.edu.cn](mailto:huanglf@xmu.edu.cn)

**Supplementary figures**


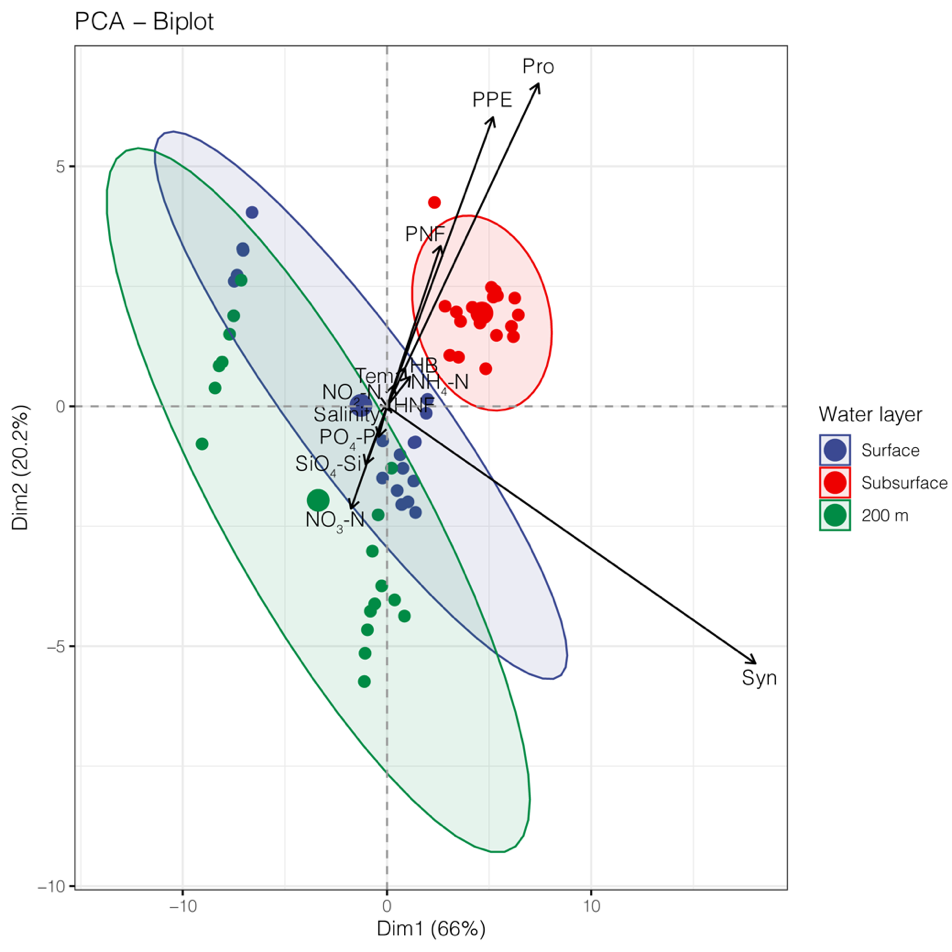


**Fig.S1** PCA ordination plot of all environmental variables in the South China Sea. The abbreviations of environmental variables are: NO_3_-N, nitrate; NO_2_-N, nitrite; NH_4_-N, ammonium; PO_4_-P, phosphate; SiO_4_-Si, silicate; HNF, heterotrophic nano-sized flagellates; PNF, pigmented nano-sized flagellates; PPE, photosynthetic picoeukaryotes; Syn*, Synechococcus*; Pro, *Prochlorococcus*; HB, heterotrophic bacteria.


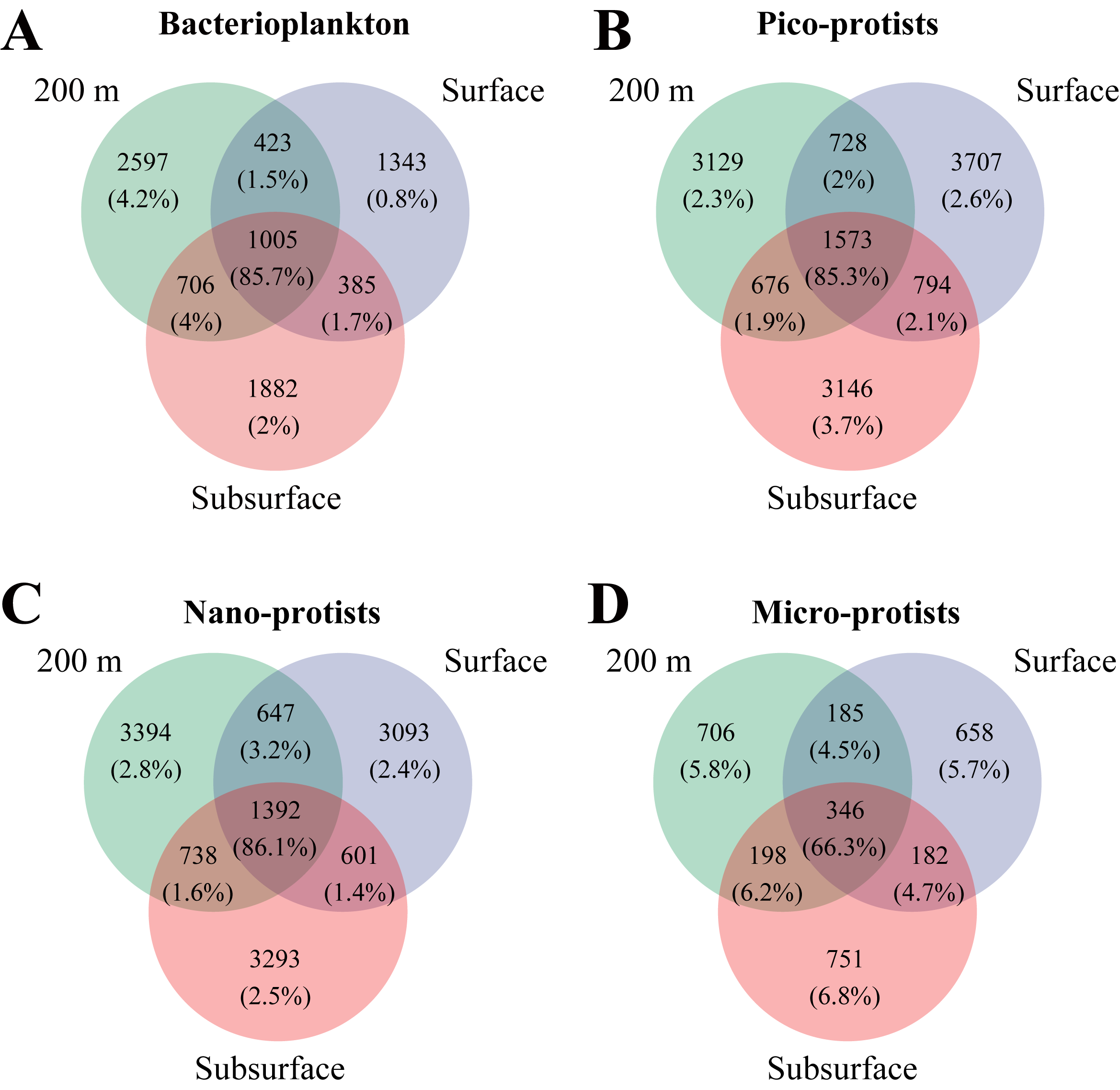


**Fig.S2** Venn diagram showing the number and percentages of bacterial and three size fractions of microbial eukaryotic ASVs that are unique and shared among three water layers. The integer is ASV number, the percentage data is the sequence number/total sequence number.


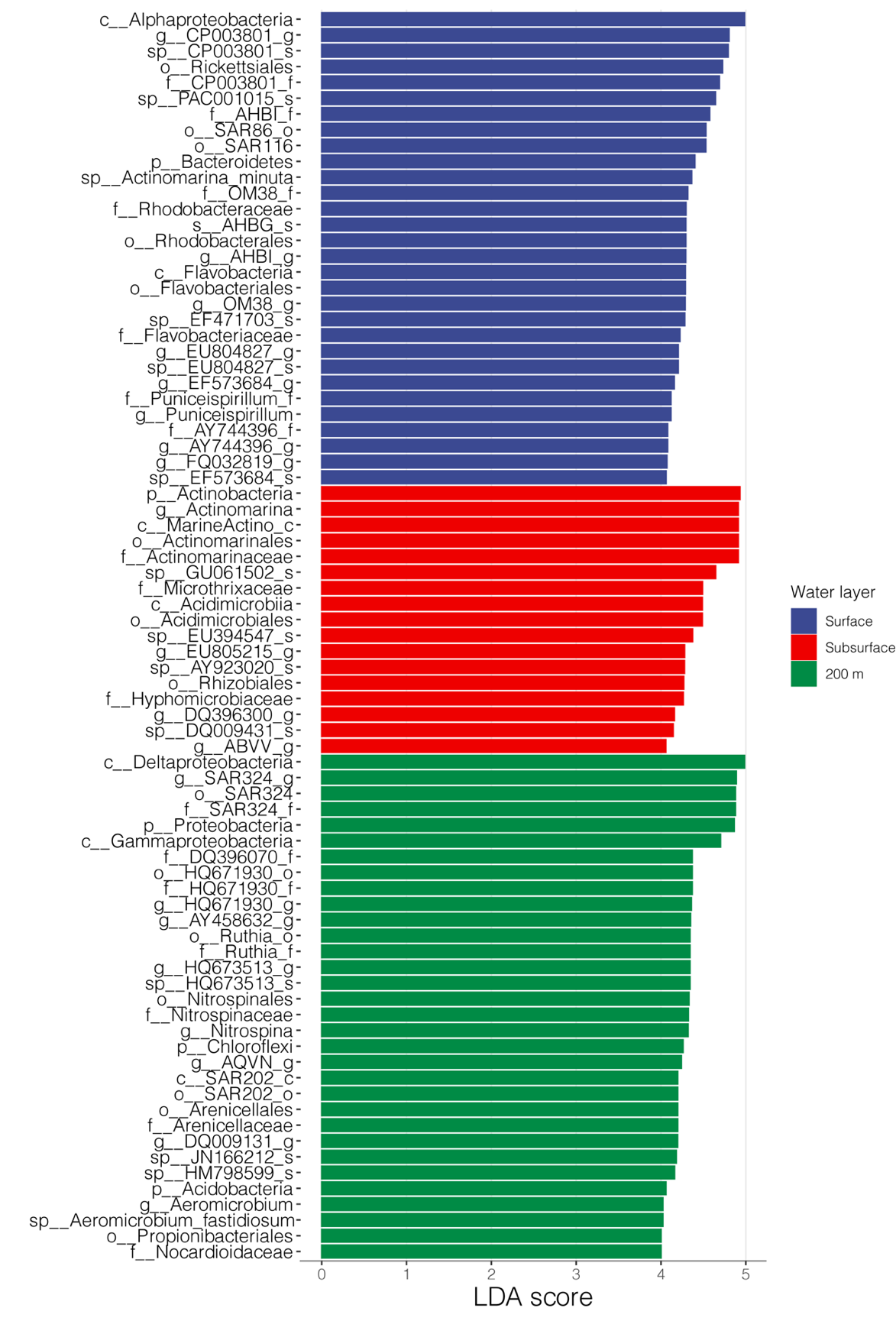


**Fig.S3** LEfSe histogram (LDA score > 4.0) showing differential bacterial taxa in three water layers. The prefix of the taxon name represents the level of taxonomy: p, phylum; c, class; o, order; f, family; g, genus; sp, species.


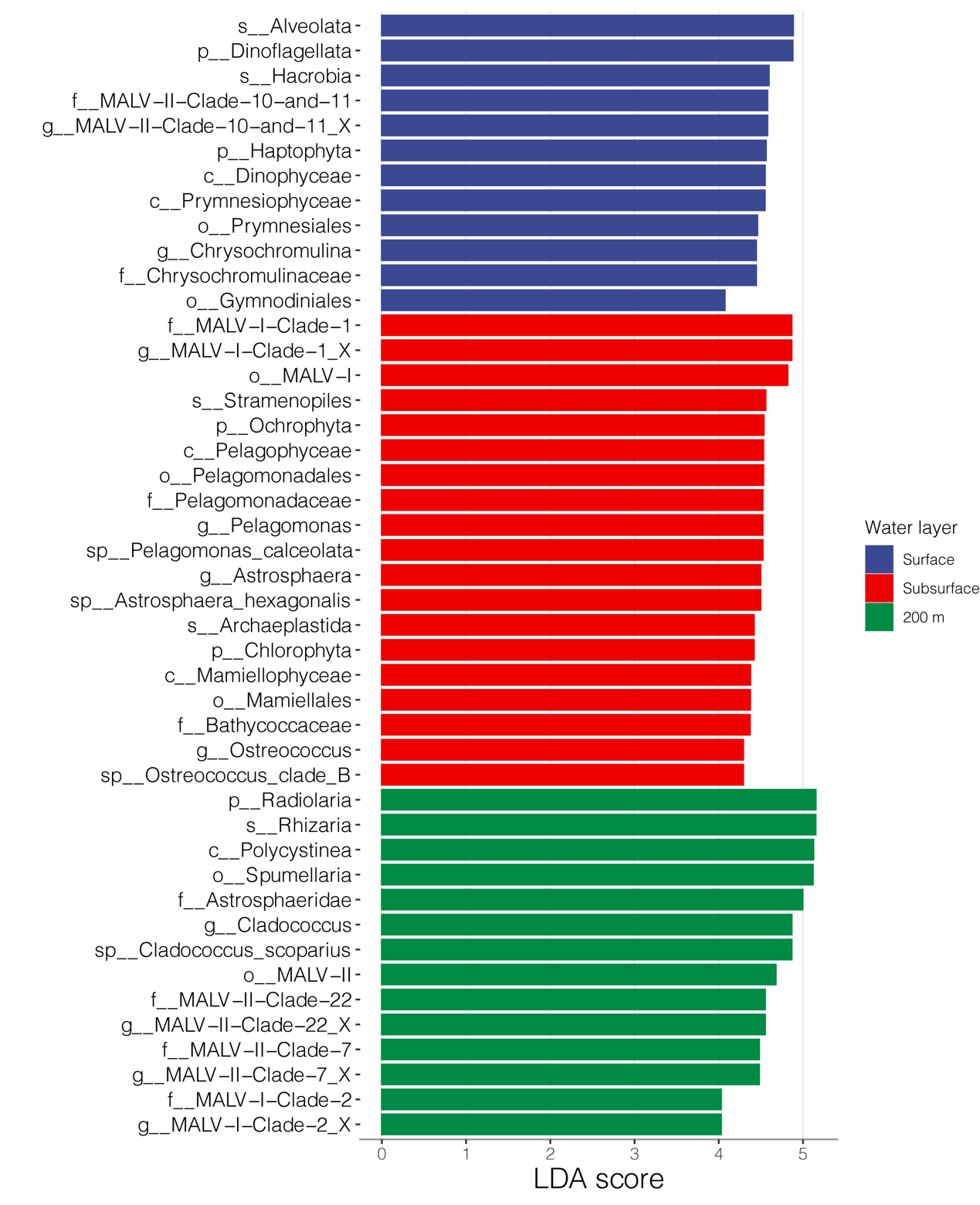


**Fig.****S4** LEfSe histogram (LDA score > 4.0) showing differential pico-protist taxa in three water layers. The taxon name's prefix represents the taxonomy level: s, supergroup; p, phylum; c, class; o, order; f, family; g, genus; sp, species.


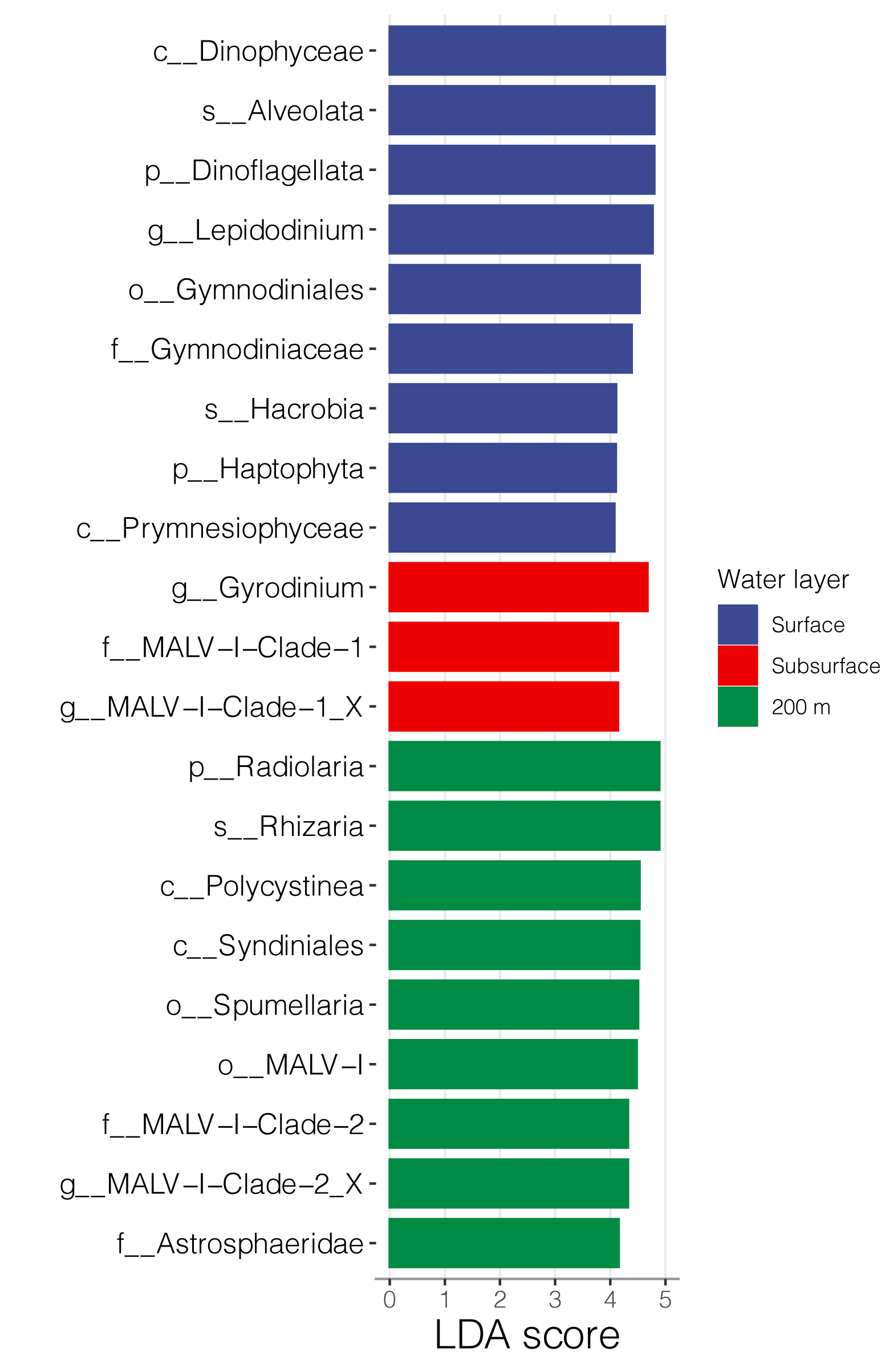


**Fig.S5** LEfSe histogram (LDA score > 4.0) showing differential nano-protist taxa in three water layers. The taxon name's prefix represents the taxonomy level, as described in the caption of supplementary Fig.S5.


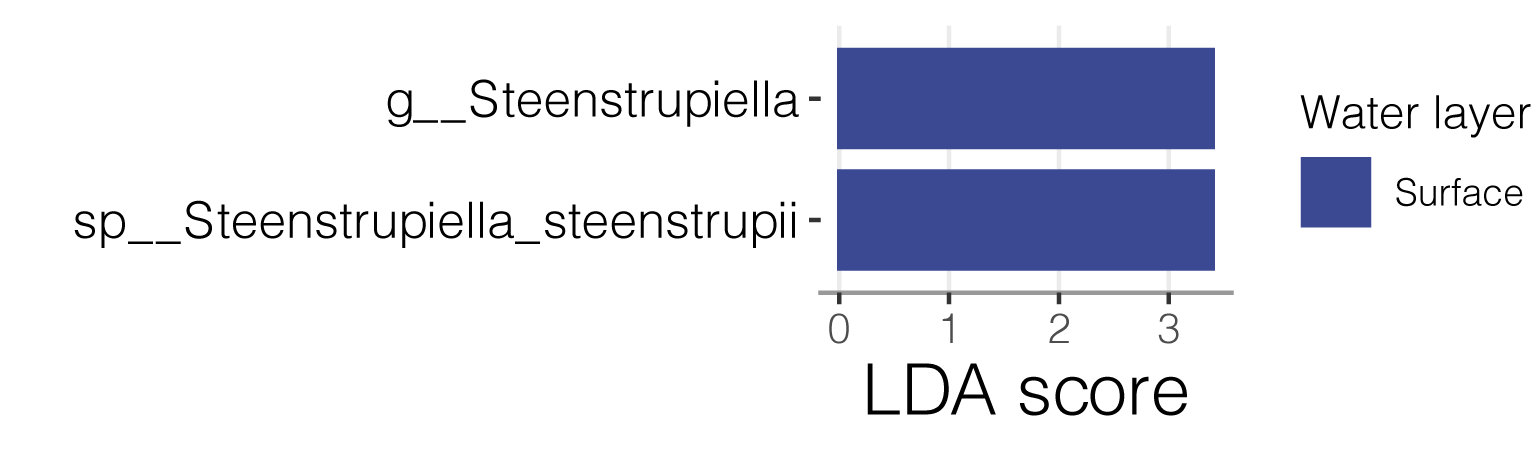


**Fig.S6** LEfSe histogram (LDA score > 3.0) showing differential micro-protist taxa in three water layers. The taxon name's prefix represents the taxonomy level, as described in the caption of supplementary Fig.S5.


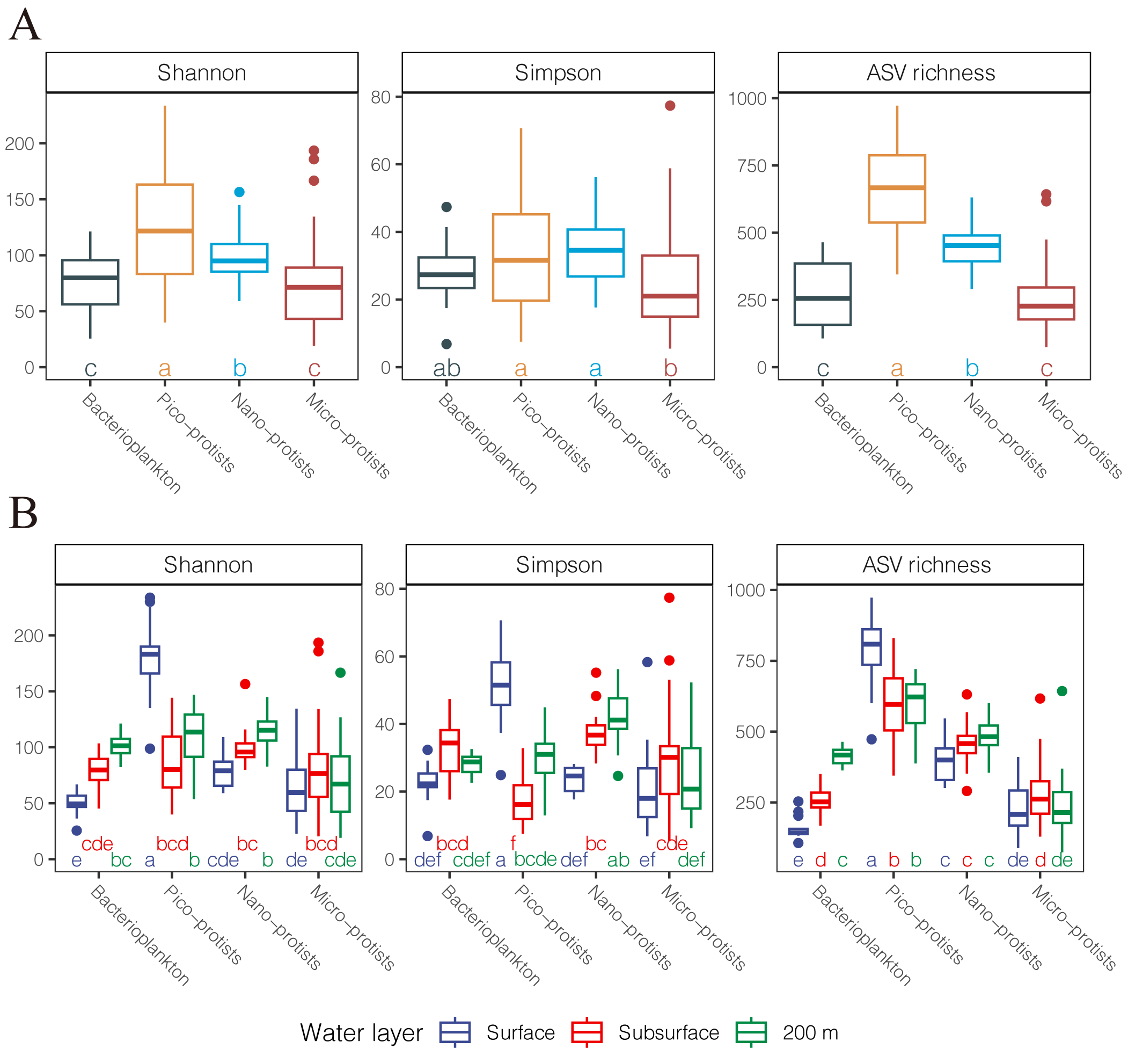


**Fig.S7** Boxplots of α-diversity indices of microbial (A) metacommunities and (B) communities in surface, subsurface, and 200 m water layers. α-diversity including species richness, Shannon's diversity and Simpson's diversity was calculated at fixed coverage (90.5%). Metacommunities are defined as the collective of bacteria and three size fractions of microbial protists. Multiple comparisons (LSD tests) are calculated among water layers. Letters indicate a significant difference (*p* < 0.05).


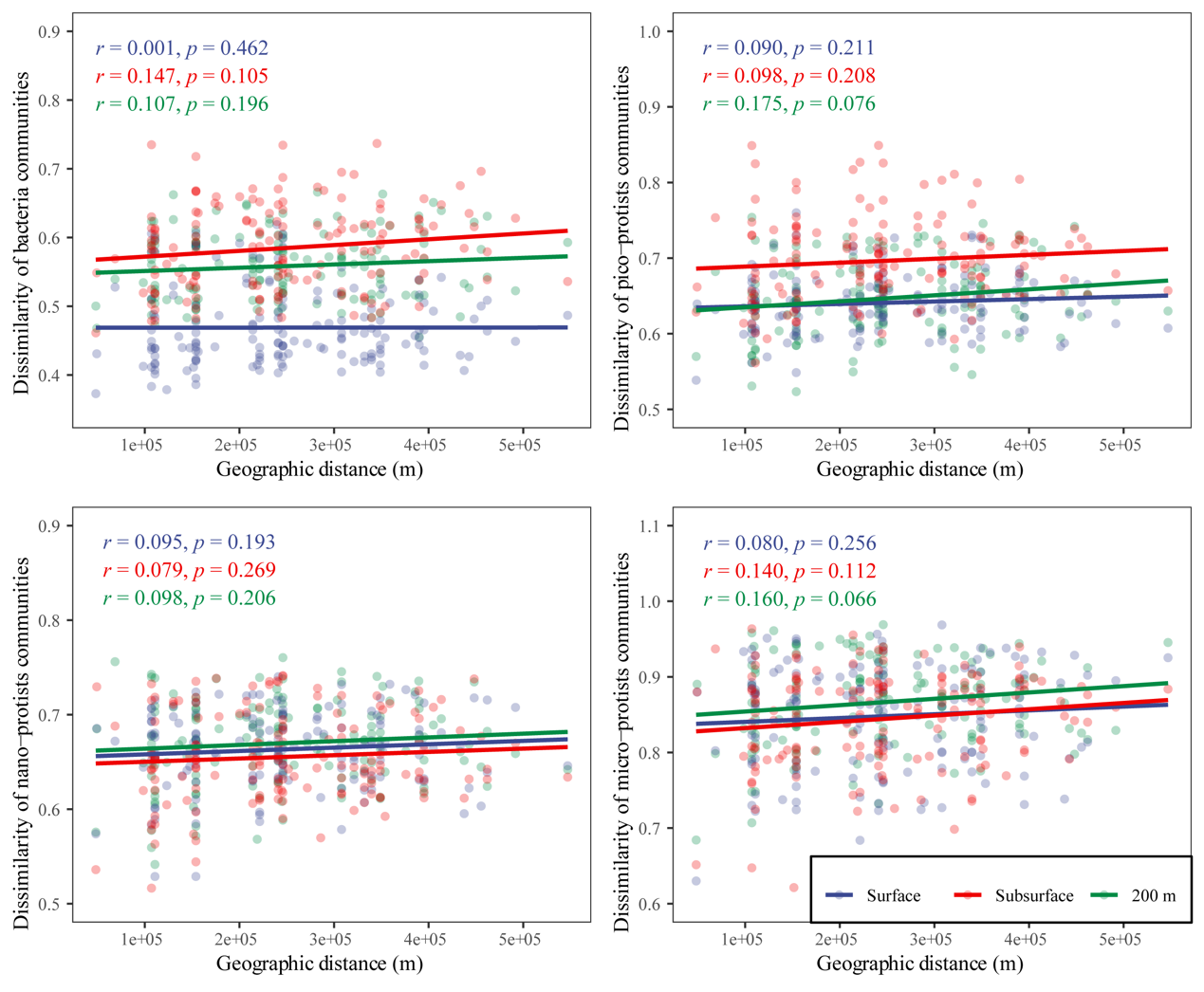


**Fig****.S8** Distance-decay patterns showing the correlations between the Bray-Curtis dissimilarity of microbial communities and geographic distance between pairs of stations in surface, subsurface, and 200 m water layers. Lines represent linear fits. The correlation coefficient (*r*) and *p*-value represent Pearson’s rank correlation based on Mantel tests.


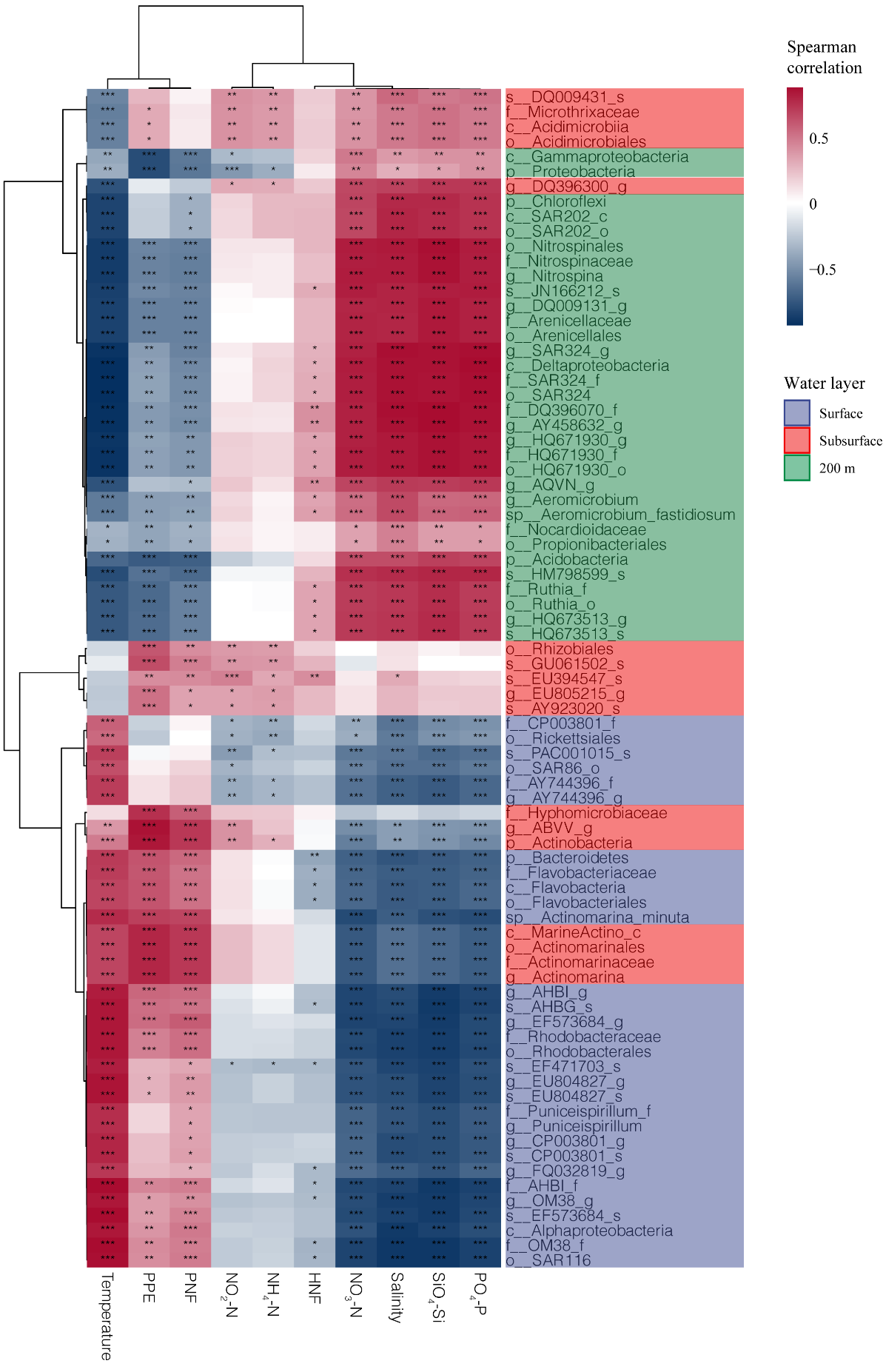


**Fig.S9** Heatmap depicting Spearman’s rank correlations between selected bacterioplankton (LDA score > 4.0) and environmental factors. The taxon name's prefix represents the taxonomy level, as described in the caption of supplementary Fig.S5. The coloring of the taxon name represents the most abundant water layer for the taxonomy. Asterisk indicates significant differences at the level of *p* < 0.05 (*, *p* < 0.05; **, *p* < 0.01; ***, *p* < 0.001).


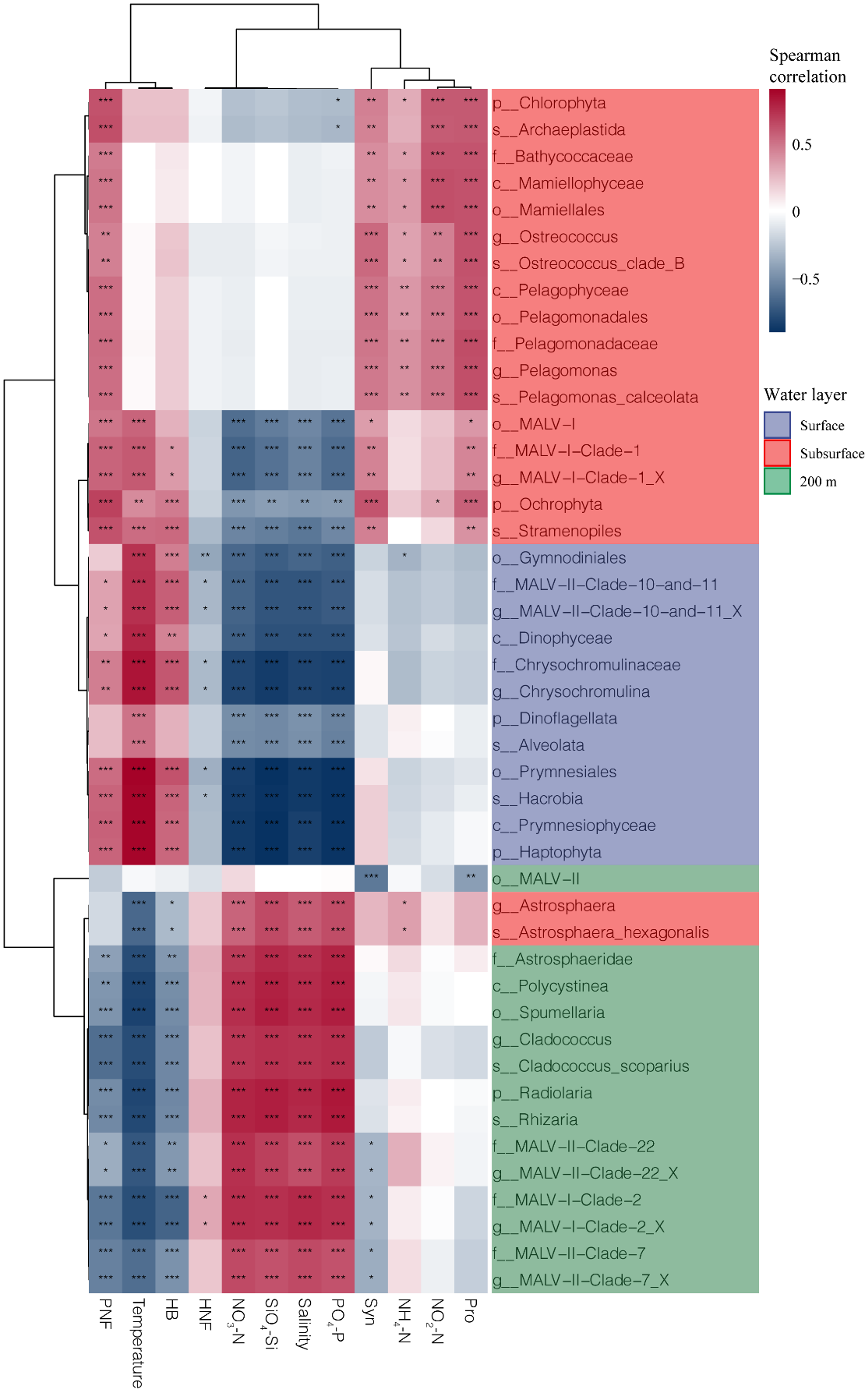


**Fig.S10** Heatmap depicting Spearman’s rank correlations between selected pico-protists (LDA score > 4.0) and environmental factors. The taxon name's prefix represents the taxonomy level, as described in the caption of supplementary Fig.S5. The coloring of the taxon name represents the most abundant water layer for the taxonomy. Asterisk indicates significant differences at the level of *p* < 0.05 (*, *p* < 0.05; **, *p* < 0.01; ***, *p* < 0.001).


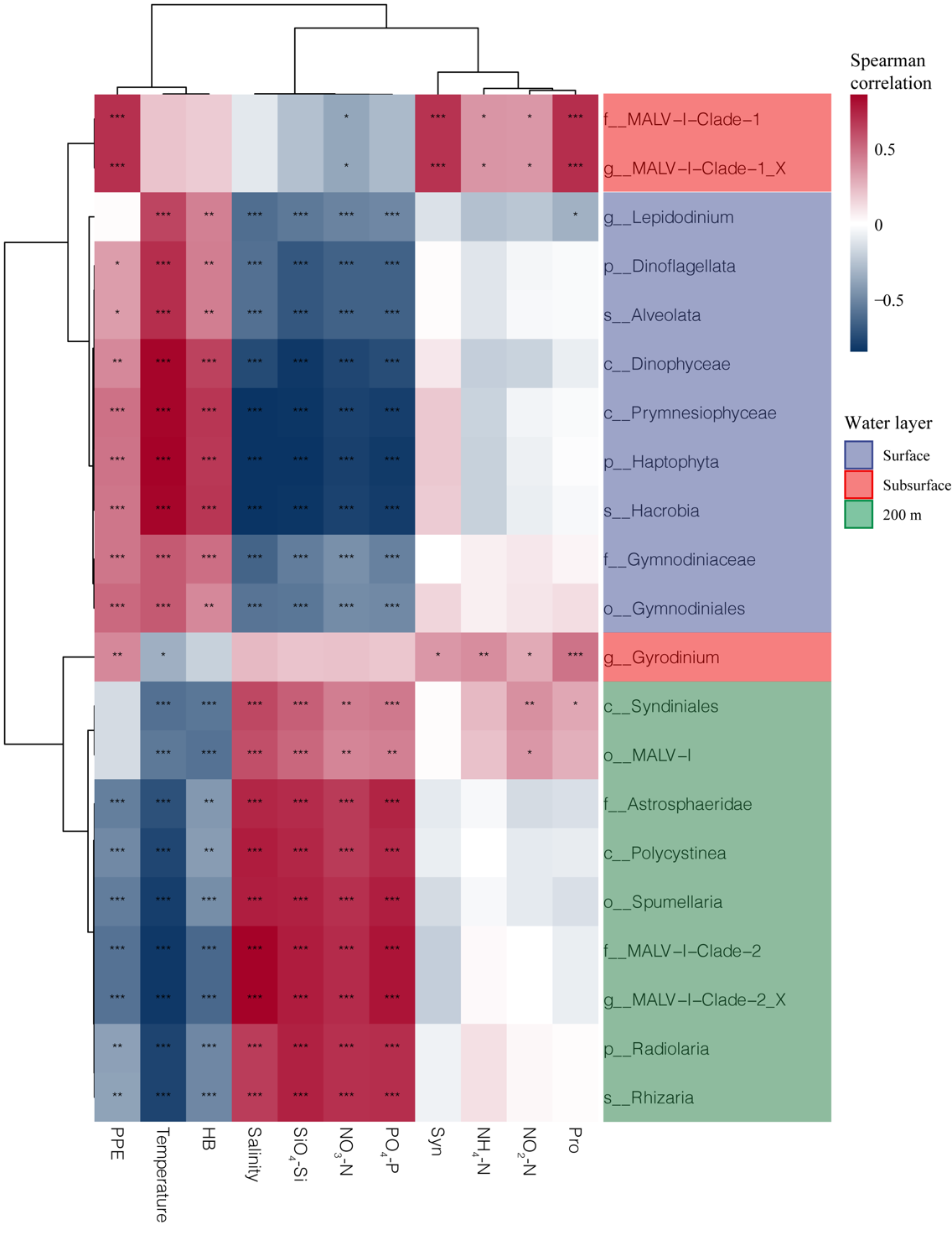


**Fig.S11** Heatmap depicting Spearman’s rank correlations between selected nano-protists (LDA score > 4.0) and environmental factors. The taxon name's prefix represents the taxonomy level, as described in the caption of supplementary Fig.S5. The coloring of the taxon name represents the most abundant water layer for the taxonomy. Asterisk indicates significant differences at the level of *p* < 0.05 (*, *p* < 0.05; **, *p* < 0.01; ***, *p* < 0.001).


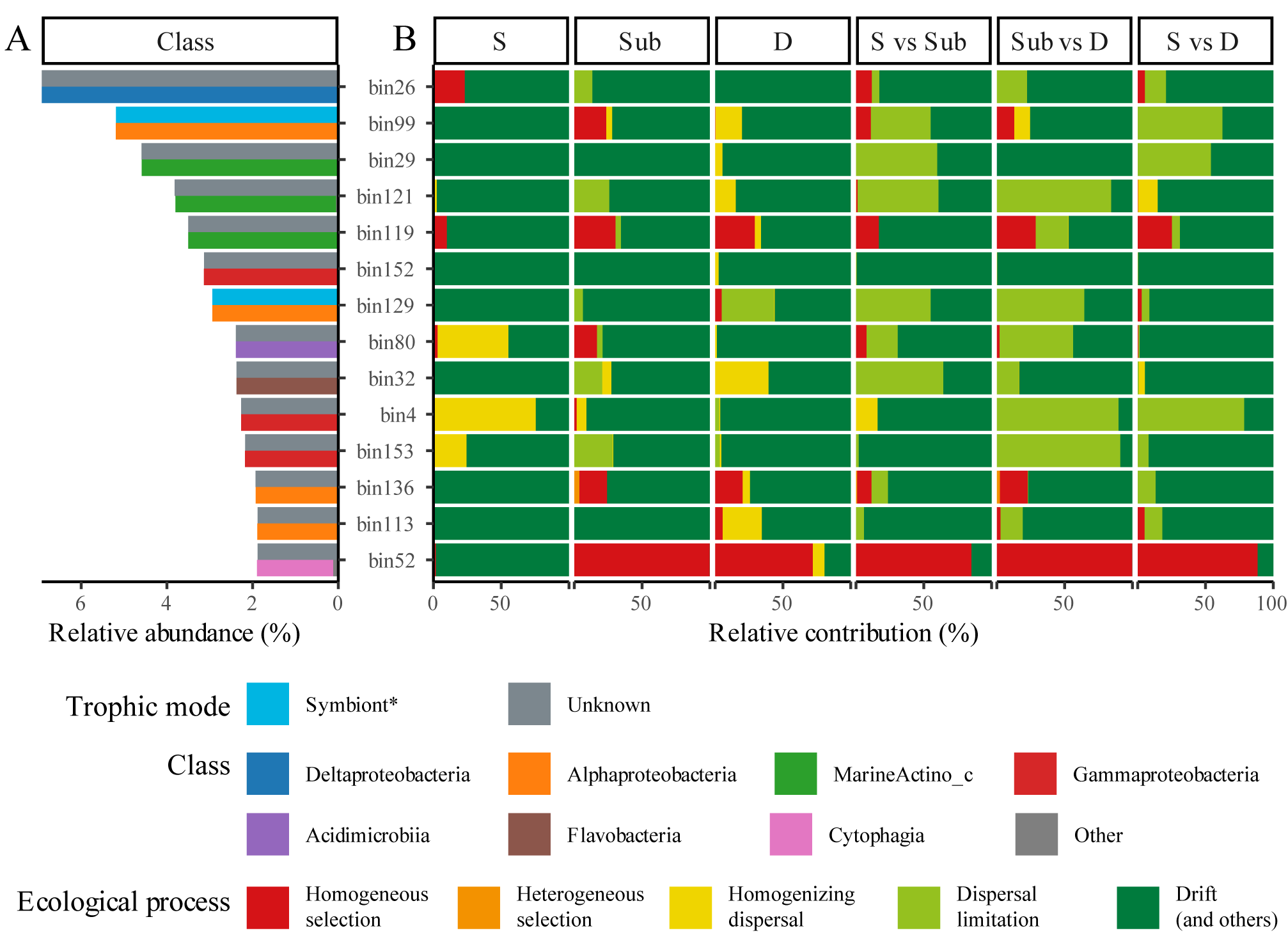


**Fig.S12** Variations in (A) relative abundance at trophic modes, class level, and (B) ecological processes of major bacterioplankton phylogenetic groups (bins). Ecological process of bacterioplankton communities within each water layer and between water layers based on inference of community assembly mechanisms by phylogenetic bin (iCAMP). The abbreviations of water layers are: S, surface; Sub, subsurface; D, 200 m. Notes: Symbiont* in bacterioplankton community are the assemblages of symbiotic, parasitic, and pathogenic taxa.


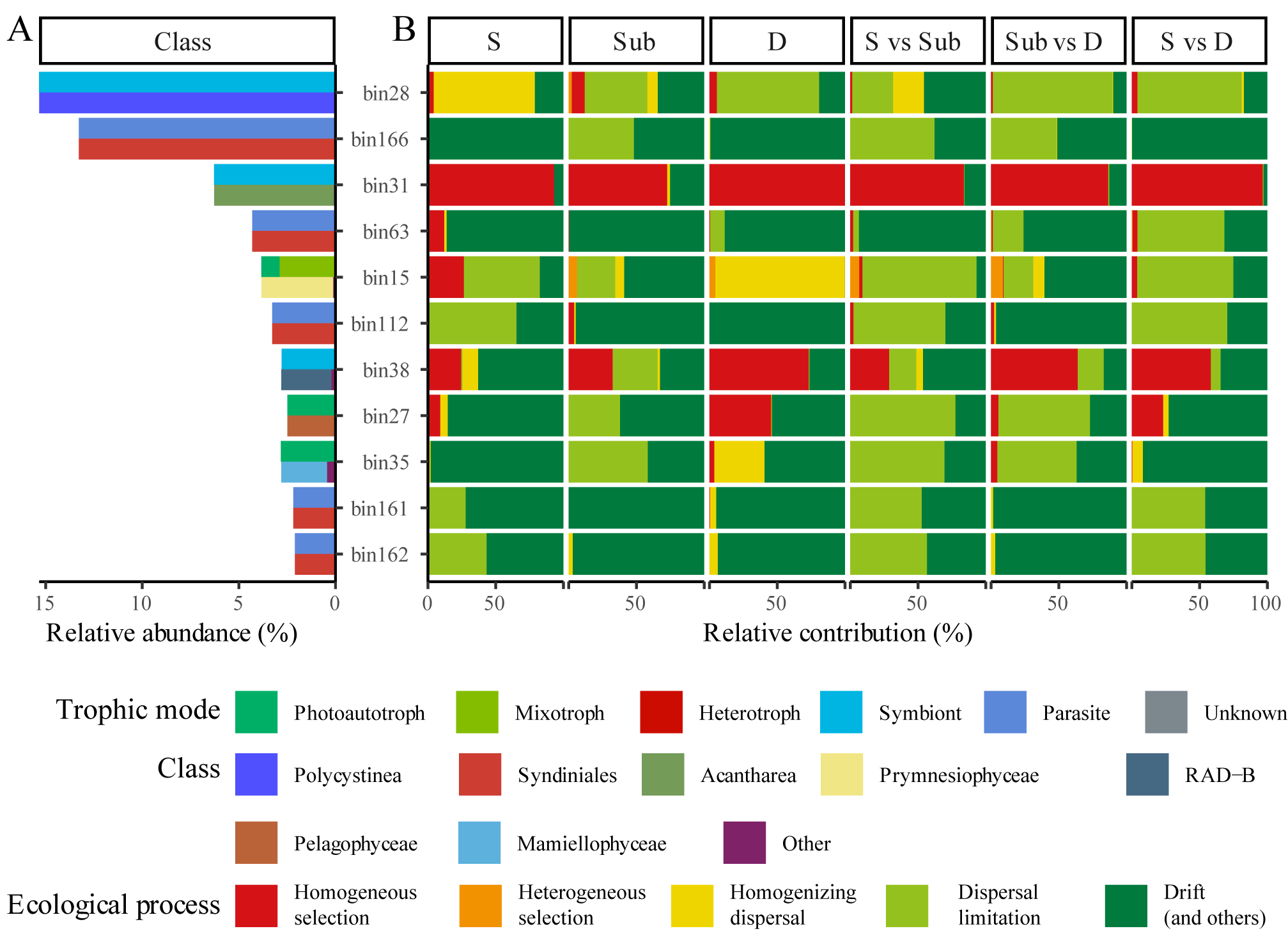


**Fig.S13** Variations in (A) relative abundance at trophic modes, class level, and (B) ecological processes of major pico-protists phylogenetic groups. Ecological process of pico-protist communities within each water layer and between water layers based on inference of community assembly mechanisms by phylogenetic bin (iCAMP). The abbreviations of water layers are: S, surface; Sub, subsurface; D, 200 m.


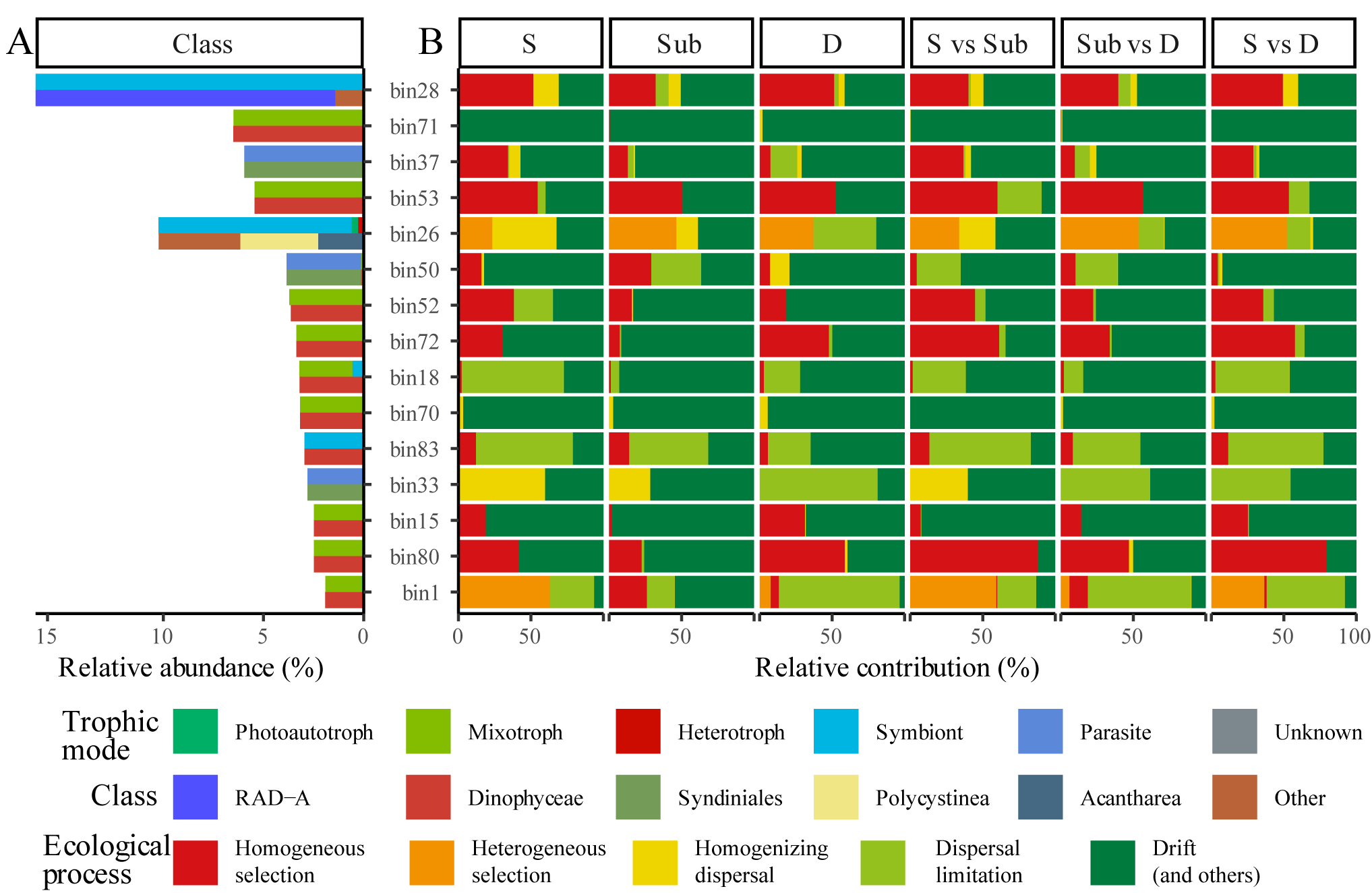


**Fig.S14** Variations in (A) relative abundance at trophic modes, class level, and (B) ecological processes of major nano-protists phylogenetic groups. Ecological process of nano-protist communities within each water layer and between water layers based on inference of community assembly mechanisms by phylogenetic bin (iCAMP). The abbreviations of water layers are: S, surface; Sub, subsurface; D, 200 m.


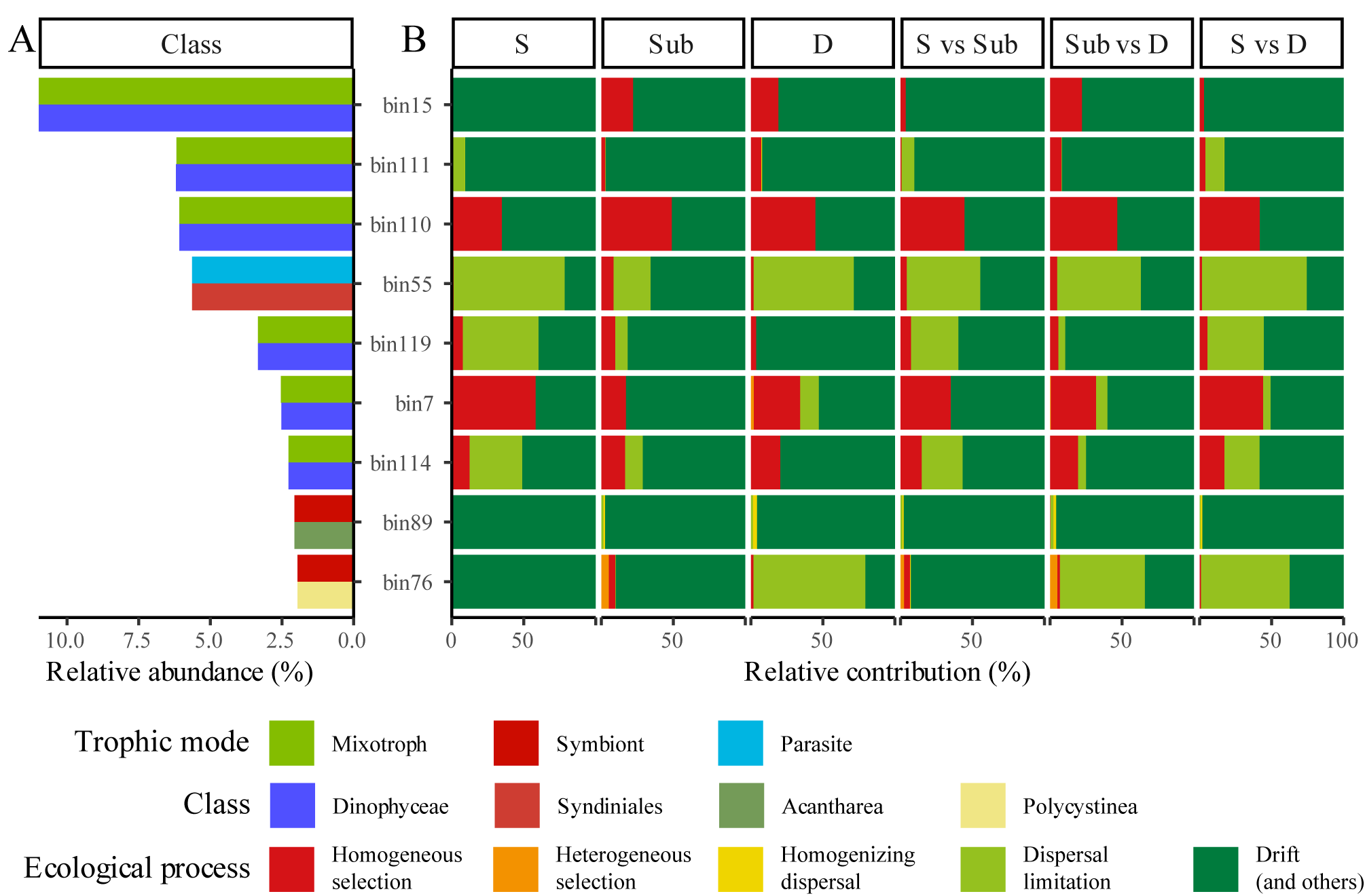


**Fig.S15** Variations in (A) relative abundance at trophic modes, dclass level, and (B) ecological processes of major micro-protists phylogenetic groups. Ecological process of micro-protist communities within each water layer and between water layers based on inference of community assembly mechanisms by phylogenetic bin (iCAMP). The abbreviations of water layers are: S, surface; Sub, subsurface; D, 200 m.


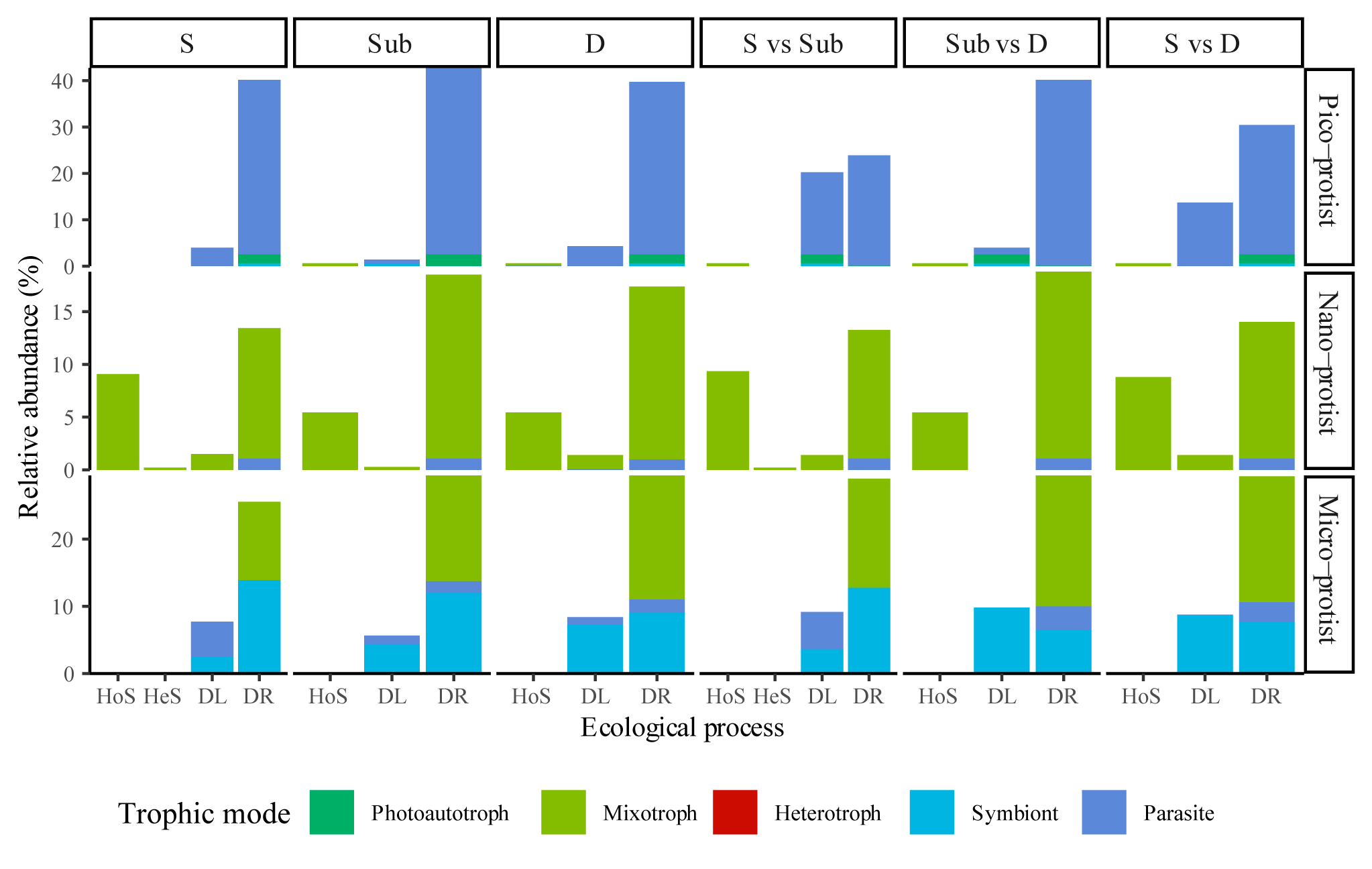


**Fig.S16**  Relative abundance of trophic modes of bins consisting of a single trophic mode and their corresponding dominant ecological processes. The abbreviations of water layers are: S, surface; Sub, subsurface; D, 200 m. The abbreviations of ecological processes are: HoS, homogenous selection;HeS, heterogeneous selection; DL, dispersal limiation; DR, drift and other (including diversification, weak selection, and/or weak dispersal).

**Supplementary tables**

**Table S1** Environmental factors at different water layers.

|  | Surface | Subsurface | 200 m |
| --- | --- | --- | --- |
| Temperature  (℃) | 30.21 ± 0.43 | 23.1 ± 1.17 | 15.51 ± 1 |
| Salinity  (psu) | 33.65 ± 0.33 | 34.39 ± 0.13 | 34.59 ± 0.03 |
| NO_3_-N  (μmol/L) | 0.24 ± 0.18 | 1.99 ± 1.87 | 13.57 ± 2.23 |
| NO_2_-N  (μmol/L) | 0.03 ± 0.01 | 0.07 ± 0.04 | 0.04 ± 0.04 |
| NH_4_-N  (μmol/L) | 1.14 ± 2.47 | 2.99 ± 2.2 | 1.37 ± 2.15 |
| PO_4_-P  (μmol/L) | 0.02 ± 0.01 | 0.13 ± 0.12 | 0.77 ± 0.19 |
| SiO_3_-Si  (μmol/L) | 2.78 ± 0.51 | 5.81 ± 2.45 | 17.38 ± 3.07 |
| HNF  (cells/mL) | 248 ± 83 | 315 ± 114 | 348 ± 103 |
| PNF  (cells/mL) | 393 ± 171 | 667 ± 273 | 80 ± 131 |
| PPE  (cells/mL) | 42978 ± 10849 | 264767 ± 154922 | 4377 ± 4413 |
| Syn  (cells/mL) | 8004 ± 6929 | 88215 ± 77913 | 6379 ± 8193 |
| Pro  (cells/mL) | 71600 ± 86895 | 8575000 ± 3919229 | 117289 ± 120958 |
| HB  (cells/mL) | 35741176 ± 12981095 | 23933333 ± 14400299 | 8562778 ± 2390547 |

The abbreviations of environmental variables are: NO_3_-N, nitrate; NO_2_-N, nitrite; NH_4_-N, ammonium; PO_4_-P, phosphate; SiO_4_-Si, silicate; HNF, heterotrophic nano-sized flagellates; PNF, pigmented nano-sized flagellates; PPE, photosynthetic picoeukaryotes; Syn*, Synechococcus*; Pro, *Prochlorococcus*; HB, heterotrophic bacteria.

**Table S2** PERMANOVA based on Bray-Curtis dissimilarity of microbial communities among three water layers.

| Community | Pairs of water layers | *R*^2^ | *p*_adjusted_ |
| --- | --- | --- | --- |
| Bacterioplankton | Surface vs Subsurface | 0.309 | 0.001 |
|  | Surface vs 200 m | 0.416 | 0.001 |
|  | Subsurface vs 200 m | 0.304 | 0.001 |
| Pico-protists | Surface vs Subsurface | 0.129 | 0.001 |
|  | Surface vs 200 m | 0.165 | 0.001 |
|  | Subsurface vs 200 m | 0.114 | 0.001 |
| Nano-protists | Surface vs Subsurface | 0.102 | 0.001 |
|  | Surface vs 200 m | 0.098 | 0.001 |
|  | Subsurface vs 200 m | 0.061 | 0.001 |
| Micro-protists | Surface vs Subsurface | 0.037 | 0.054 |
|  | Surface vs 200 m | 0.044 | 0.012 |
|  | Subsurface vs 200 m | 0.036 | 0.054 |

**Table S3** Comparison of key ecological processes between each group based on the bootstrapping test.

| Community | Group1 | Group2 | HoS  Cohen.d | HoS  Effect Size | DL  Cohen.d | DL  Effect Size | DR  Cohen.d | DR  Effect Size |
| --- | --- | --- | --- | --- | --- | --- | --- | --- |
| Bacterioplankton | D | S | 2.23 | L | 6.70 | **L** | -7.76 | **L** |
|  | D | Sub | -2.57 | **L** | 1.18 | L | -0.69 | M |
|  | D | S vs D | 1.30 | L | -7.41 | **L** | 6.69 | **L** |
|  | D | Sub vs D | 0.21 | S | -12.57 | **L** | 13.28 | **L** |
|  | D | S vs Sub | -2.65 | **L** | -5.11 | **L** | 6.54 | **L** |
|  | S | Sub | -3.66 | **L** | -5.93 | **L** | 6.65 | **L** |
|  | S | S vs D | -1.22 | L | -20.16 | **L** | 15.01 | **L** |
|  | S | Sub vs D | -2.15 | L | -27.60 | **L** | 22.16 | **L** |
|  | S | S vs Sub | -3.72 | **L** | -16.12 | **L** | 15.69 | **L** |
|  | Sub | S vs D | 3.21 | **L** | -9.56 | **L** | 7.04 | **L** |
|  | Sub | Sub vs D | 2.75 | **L** | -15.21 | **L** | 13.23 | **L** |
|  | Sub | S vs Sub | -0.09 | N | -6.98 | **L** | 6.89 | **L** |
|  | S vs D | Sub vs D | -1.18 | L | -6.55 | **L** | 6.68 | **L** |
|  | S vs D | S vs Sub | -3.27 | **L** | 2.66 | **L** | -0.85 | L |
|  | Sub vs D | S vs Sub | -2.82 | **L** | 8.97 | **L** | -8.40 | **L** |
| Pico-protists | D | S | 1.95 | L | 1.01 | L | -0.91 | L |
|  | D | Sub | 4.24 | **L** | 0.24 | S | -1.78 | L |
|  | D | S vs D | 1.29 | L | -2.23 | L | 1.99 | L |
|  | D | Sub vs D | 2.83 | **L** | -2.80 | **L** | 1.98 | L |
|  | D | S vs Sub | 4.32 | **L** | -3.19 | **L** | 2.55 | **L** |
|  | S | Sub | 2.97 | **L** | -0.87 | L | -1.43 | L |
|  | S | S vs D | -0.82 | L | -6.15 | **L** | 5.10 | **L** |
|  | S | Sub vs D | 1.02 | L | -6.09 | **L** | 4.10 | **L** |
|  | S | S vs Sub | 2.96 | **L** | -7.75 | **L** | 5.39 | **L** |
|  | Sub | S vs D | -3.69 | **L** | -2.98 | **L** | 4.23 | **L** |
|  | Sub | Sub vs D | -2.30 | L | -3.54 | **L** | 3.98 | **L** |
|  | Sub | S vs Sub | -0.90 | L | -4.10 | **L** | 4.66 | **L** |
|  | S vs D | Sub vs D | 1.92 | L | -1.12 | L | 0.29 | S |
|  | S vs D | S vs Sub | 3.93 | **L** | -1.66 | L | 1.01 | L |
|  | Sub vs D | S vs Sub | 2.10 | L | -0.29 | S | 0.57 | M |
| Nano-protists | D | S | -1.06 | L | 2.15 | L | 0.15 | N |
|  | D | Sub | 1.93 | L | 6.43 | **L** | -5.39 | **L** |
|  | D | S vs D | -1.08 | L | 1.55 | L | 0.21 | S |
|  | D | Sub vs D | 0.91 | L | 3.45 | **L** | -3.03 | **L** |
|  | D | S vs Sub | -1.81 | L | 3.04 | **L** | -0.61 | M |
|  | S | Sub | 3.20 | **L** | 5.31 | **L** | -6.05 | **L** |
|  | S | S vs D | -0.01 | N | -0.79 | M | 0.07 | N |
|  | S | Sub vs D | 2.31 | **L** | 1.67 | L | -3.63 | **L** |
|  | S | S vs Sub | -0.78 | M | 0.96 | L | -0.92 | L |
|  | Sub | S vs D | -3.24 | **L** | -6.16 | **L** | 5.99 | **L** |
|  | Sub | Sub vs D | -1.31 | L | -3.52 | **L** | 2.83 | **L** |
|  | Sub | S vs Sub | -4.10 | **L** | -4.95 | **L** | 5.37 | **L** |
|  | S vs D | Sub vs D | 2.36 | **L** | 2.45 | **L** | -3.60 | **L** |
|  | S vs D | S vs Sub | -0.78 | M | 1.87 | L | -0.95 | L |
|  | Sub vs D | S vs Sub | -3.38 | **L** | -0.93 | L | 2.81 | **L** |
| Micro-protists | D | S | 1.05 | L | 1.43 | L | -1.78 | L |
|  | D | Sub | -0.94 | L | 2.22 | L | -1.81 | L |
|  | D | S vs D | 1.33 | L | 0.82 | L | -1.29 | L |
|  | D | Sub vs D | -0.94 | L | 1.36 | L | -0.93 | L |
|  | D | S vs Sub | 1.27 | L | 2.03 | L | -2.41 | **L** |
|  | S | Sub | -1.66 | L | 0.79 | M | -0.05 | N |
|  | S | S vs D | 0.16 | N | -0.81 | L | 0.75 | M |
|  | S | Sub vs D | -1.95 | L | -0.23 | S | 1.07 | L |
|  | S | S vs Sub | 0.12 | N | 0.40 | S | -0.45 | S |
|  | Sub | S vs D | 1.83 | L | -1.70 | L | 0.80 | M |
|  | Sub | Sub vs D | 0.24 | S | -1.10 | L | 1.10 | L |
|  | Sub | S vs Sub | 1.80 | L | -0.49 | S | -0.38 | S |
|  | S vs D | Sub vs D | -2.27 | **L** | 0.66 | M | 0.39 | S |
|  | S vs D | S vs Sub | -0.04 | N | 1.43 | L | -1.39 | L |
|  | Sub vs D | S vs Sub | 2.20 | L | 0.73 | M | -1.72 | L |

The abbreviations of groups are: S, surface; Sub, subsurface; D, 200 m. The abbreviations of ecological processes are: HoS, homogenous selection; DL, dispersal limiation; DR, drift and other (including diversification, weak selection, and/or weak dispersal). Effect size magnitude according to Cohen's d, the abbreviations of effect size are: L, large (|d| > 0.8); M, medium (0.5 < |d| ≤ 0.8); S, small (0.2 < |d| ≤ 0.5); N, negligible (|d| ≤ 0.2). Bolded impact effect size represent p-value from bootstrapping test less than 0.05.

**Table S4** Mantel and partial Mantel tests for the correlation between environmental variables or microbial community similarity and environmental variables using Spearman’s coefficient.

| Effects of | Controlling for | Salinity | Depth | Bacterio-plankton | Pico-protists | Nano-protists | Micro-protists |
| --- | --- | --- | --- | --- | --- | --- | --- |
| Temperature |  | 0.52  *** | 0.95  *** | 0.87  *** | 0.52  *** | 0.36  *** | 0.11  ** |
| Salinity |  |  | 0.53 | 0.43  *** | 0.38  *** | 0.38  *** | 0.04 |
| Depth |  |  |  | 0.85  *** | 0.53  *** | 0.35  *** | 0.11  ** |
| Temperature | Depth | 0.06 |  | 0.34  *** | 0.09  * | 0.09  * | 0.02 |
| Salinity | Depth |  |  | -0.06 | 0.14  * | 0.25  ** | -0.02 |

*, *p* < 0.05; **, *p* < 0.01; ***, *p* < 0.001.
